# 3D autofluorescence imaging of hydronephrosis and renal anatomical structure using cryo-micro-optical sectioning tomography

**DOI:** 10.7150/thno.86695

**Published:** 2023-09-04

**Authors:** Guoqing Fan, Chenyu Jiang, Zhuoyao Huang, Mingyu Tian, Huijuan Pan, Yaru Cao, Tian Lei, Qingming Luo, Jing Yuan

**Affiliations:** 1 Britton Chance Center for Biomedical Photonics, Wuhan National Laboratory for Optoelectronics, MoE Key Laboratory for Biomedical Photonics, School of Engineering Sciences, Innovation Institute, Huazhong University of Science and Technology, Wuhan 430074, China.; 2 Research Unit of Multimodal Cross Scale Neural Signal Detection and Imaging, Chinese Academy of Medical Sciences, HUST-Suzhou Institute for Brainmatics, JITRI, Suzhou 215123, China.; 3 School of Biomedical Engineering, Hainan University, Haikou, 570228, China.

**Keywords:** hydronephrosis, UUO, db/db mice, autofluorescence imaging, cryo-imaging

## Abstract

**Rationale:** Mesoscopic visualization of the main anatomical structures of the whole kidney *in vivo* plays an important role in the pathological diagnosis and exploration of the etiology of hydronephrosis. However, traditional imaging methods cannot achieve whole-kidney imaging with micron resolution under conditions representing *in vivo* perfusion.

**Methods:** We used *in vivo* cryofixation (IVCF) to fix acute obstructive hydronephrosis (unilateral ureteral obstruction, UUO), chronic spontaneous hydronephrosis (db/db mice), and their control mouse kidneys for cryo-micro-optical sectioning tomography (cryo-MOST) autofluorescence imaging. We quantitatively assessed the kidney-wide pathological changes in the main anatomical structures, including hydronephrosis, renal subregions, arteries, veins, glomeruli, renal tubules, and peritubular functional capillaries.

**Results:** By comparison with microcomputed tomography imaging, we confirmed that IVCF can maintain the status of the kidney *in vivo*. Cryo-MOST autofluorescence imaging can display the main renal anatomical structures with a cellular resolution without contrast agents. The hydronephrosis volume reached 26.11 ± 6.00 mm^3^ and 13.01 ± 3.74 mm^3^ in 3 days after UUO and in 15-week-old db/db mouse kidneys, respectively. The volume of the cortex and inner stripe of the outer medulla (ISOM) increased while that of the inner medulla (IM) decreased in UUO mouse kidneys. Db/db mice also showed an increase in the volume of the cortex and ISOM volume but no atrophy in the IM. The diameter of the proximal convoluted tubule and proximal straight tubule increased in both UUO and db/db mouse kidneys, indicating that proximal tubules were damaged. However, some renal tubules showed abnormal central bulge highlighting in the UUO mice, but the morphology of renal tubules was normal in the db/db mice, suggesting differences in the pathology and severity of hydronephrosis between the two models. UUO mouse kidneys also showed vascular damage, including segmental artery and vein atrophy and arcuate vein dilation, and the density of peritubular functional capillaries in the cortex and IM was reduced by 37.2% and 49.5%, respectively, suggesting renal hypoxia. In contrast, db/db mouse kidneys showed a normal vascular morphology and peritubular functional capillary density. Finally, we found that the db/db mice displayed vesicoureteral reflux and bladder overactivity, which may be the cause of hydronephrosis formation.

**Conclusions:** We observed and compared main renal structural changes in hydronephrosis under conditions representing *in vivo* perfusion in UUO, db/db, and control mice through cryo-MOST autofluorescence imaging. The results indicate that cryo-MOST with IVCF can serve as a simple and powerful tool to quantitatively evaluate the *in vivo* pathological changes in three dimensions, especially the distribution of body fluids in the whole kidney. This method is potentially applicable to the three-dimensional visualization of other tissues, organs, and even the whole body, which may provide new insights into pathological changes in diseases.

## Introduction

Hydronephrosis is a common clinical disease. Long-term hydronephrosis may cause renal medullary atrophy, renal fibrosis, and inflammation and even lead to renal failure 1. Numerous etiologies can induce hydronephrosis, such as urinary tract obstruction, vesicoureteral reflux, bladder dysfunction, etc. 1-3. Different etiologies and grades of hydronephrosis lead to different changes in renal anatomical structures and affect the choice of treatment 4-6. Therefore, observing the distribution of hydronephrosis and the mesoscopic anatomical structures of the whole kidney *in situ* is helpful for identifying disease, selecting treatment, and evaluating therapeutic efficacy.

Methods for imaging hydronephrosis and the renal parenchyma in model animals include *in vivo* imaging and *ex vivo* imaging. Ultrasound 7, magnetic resonance imaging 8, and computed tomography (CT) 9 can be used to directly observe the real-time status of the kidney *in vivo*. However, these methods have difficulty distinguishing cellular anatomical structures, which are limited by unsatisfactory resolution. In addition, the use of exogenous contrast agents increases the contrast of their images but also risks altering renal hemodynamics and causing changes in vasculature 10, 11. Multiphoton microscopy can be used to visualize the dynamic processes of renal structures at the cellular level, but the imaging depth and speed are insufficient to realize whole-kidney imaging 12, 13. *Ex vivo* renal imaging techniques, including optical imaging of tissue sections 14, 15, light sheet microscopy combined with optical clearing 16, and microcomputed tomography (micro-CT) imaging 17, 18, have improved our understanding of the three-dimensional (3D) morphology of the kidney. However, the excision and fixation of the kidney changes the *in vivo* hydronephrosis and blood perfusion status. Therefore, there is no convenient technique to obtain both an accurate 3D distribution of hydronephrosis and mesoscopic anatomical details *in vivo* with cellular resolution throughout the kidney.

Cryo-imaging technology can be used to obtain mesoscopic images of frozen tissue, organs, or even the whole body in a low-temperature environment 19-21. Freezing preserves the morphology of the biological sample *in situ*. The imaging depth is not limited by removing the imaged tissue mechanically. David L. Wilson et al. observed the distribution of stem cells 22 in a whole mouse using their Cryoviz system. Athanasios Sarantopoulos et al. developed cryo-slicing imaging technology to obtain the distribution of fluorescent probes in a whole mouse 23. However, most of these studies use specific markers or contrast agents, and the spatial resolution limitations make it difficult to observe microscopic structures such as capillaries. Autofluorescence imaging has the potential to be widely used in the study of disease mechanisms since it can reflect structural and metabolic information of the body without external markers. One of the limitations of autofluorescence imaging is that tissue autofluorescence is weak and difficult to detect at room temperature, but fluorescence quantum efficiency increases as the temperature decreases 20, 24, 25. Therefore, autofluorescence imaging can be easily performed at low temperatures. Mahsa Ranji et al. obtained metabolic and vascular information of kidneys and lungs in normal and irradiated rats by autofluorescence imaging at -40 ℃ 26. However, this method is also limited by low spatial resolution, and renal tubules and capillaries are difficult to distinguish. Our group developed cryo-micro-optical sectioning tomography (cryo-MOST) technology to achieve autofluorescence imaging with micron resolution at liquid nitrogen temperatures 27. We used cryo-MOST to obtain the brain-wide distribution of senile plaques 27 and tissue metabolism information in acute lung injury 28 and tumor mouse models 29 without labeling. Therefore, cryo-MOST has the potential to achieve 3D kidney-wide imaging of hydronephrosis and the renal parenchyma with micron resolution.

In this study, we used an *in vivo* cryofixation (IVCF) method to maintain the *in vivo* morphology and vascular perfusion status of mouse kidneys. Unilateral ureteral obstruction (UUO) and db/db mice were chosen as acute and chronic hydronephrosis models, respectively, to perform whole-kidney cryo-MOST autofluorescence imaging with micron resolution. We obtained the volume and distribution of hydronephrosis and renal subregions in UUO and db/db mouse kidneys. The changes in mesoscopic anatomical structures, including the 3D vascular network, glomeruli, renal tubules, and functional capillaries, were also analyzed. These detailed analyses help us to understand the course of hydronephrosis and the interactions among various components during the development of the disease.

## Methods

### Cryo-MOST imaging

A schematic of the cryo-MOST imaging system is shown in Figure S1. The system consisted of optical imaging and mechanical cutting components. A halogen lamp (X-Cite exacte, Olympus, Japan) was used as the light source. A stereo microscope (MVX10, Olympus, Japan) was used due to the flexible microscopic imaging capability of the ZOOM module and long working distance longer than 10 mm. The turret filter allowed imaging of up to three fluorescence channels. The fluorescence filters used in this work included a 434 nm excitation filter (FF01-434/17, Semrock, USA), a dichroic mirror of 510 nm (FF510-Di02, Semrock, USA), and a 536 nm emission filter (FF01-536/40, Semrock, USA). The images were captured by an sCMOS camera (ORCA-Flash 4.0, Hamamatsu, Japan) at an adjustable lateral resolution from 0.52 to 5.16 μm/pix according to experimental needs.

The mechanical cutting component included a milling machine (DG003, Sheng Han Machinery, China), XYZ translation stage (X stage: M531, Y stage: M521, Z stage: HPS-170, Physik Instrumente, Germany), and a liquid nitrogen tank. The samples were embedded using a precooled embedding agent of 10% ethanol, 30% glycerol, and 60% deionized water and fixed in the liquid nitrogen tank on the XYZ translation stage. The stage was used to drive the sample back and forth between the objective lens and the milling cutter to obtain 3D images of the samples. The minimal milling thickness was 1 μm.

**Figure 1:**
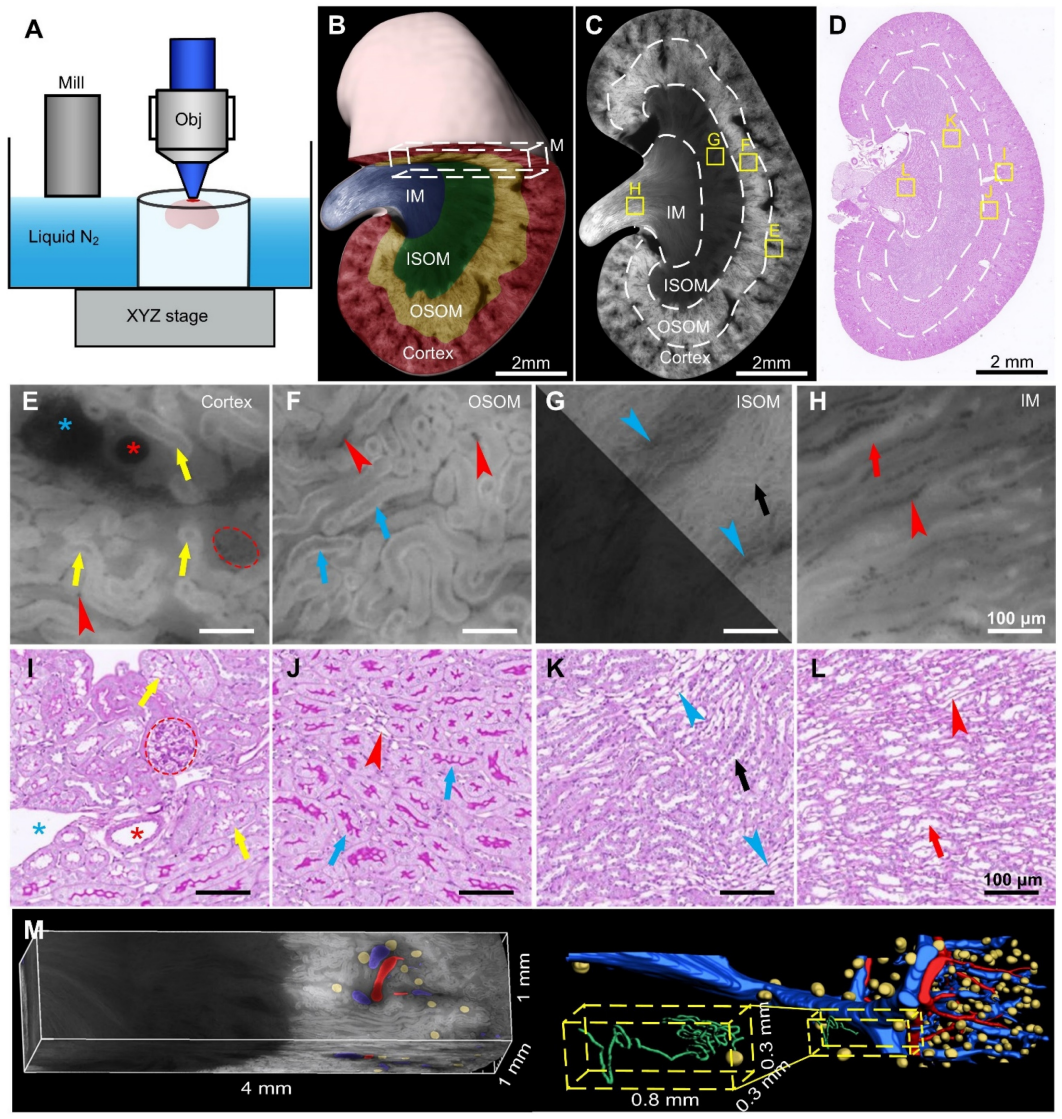
** Cryo-micro-optical sectioning tomography (cryo-MOST) imaging of normal C57 mouse kidney and its comparison with Periodic Acid-Schiff (PAS)-stained histological section.** (A) Scheme of the cryo-MOST system. (B) Three-dimensional (3D) reconstruction and cross-sectional display of 3/4 C57 mouse kidneys. Different subregions are represented by different pseudocolors. (C) Cryo-MOST image of the maximum coronal section in (B). The boundaries of different subregions are marked with white dotted lines. (D) PAS-stained histological section of another C57 mouse kidney at a similar position as in (C). (E-L) Enlarged images of the yellow squares in (C) and (D), including the cortex (E, I), outer stripe of the outer medulla (F, J), inner stripe of the outer medulla (G, K), and inner medulla (H, L). We adjusted the contrast in the upper right part of (G) to facilitate viewing. The essential microstructures of the kidney, including glomeruli (red dotted line), proximal convoluted tubules (yellow arrows), proximal straight tubules (blue arrows), Henle's loops (black arrows), collecting ducts (red arrows), arteries (red *), veins (blue *), capillaries (red arrowheads), and medullary vascular bundles (blue arrowheads), are easily identifiable. (M) 3D rendering of a 4 × 1 × 1 mm3 cuboid indicated by the white dashed box in (A) and its 3D reconstruction of arteries (red), veins (blue), glomeruli (yellow), and renal tubule (green). The inset is the enlarged view of a yellow dashed box indicating a glomerulus and its connecting renal tubule.

In this study, we used a resolution of 10 μm in the z-direction in Figure 1 and Movie 1 and 20 μm in the 3D reconstruction of renal blood vessels (Figure 4, Figure 6, Movie 2, and Movie 3). The lateral resolution was set to 4.2 μm/pix for all sections and 1.67 μm/pix for high-resolution images repeatedly captured at 200 μm intervals.

### Animals

Eight-week-old (8 W) male C57BL/6J mice were purchased from Vital River Laboratories (Beijing, China). All 6-week-old (6 W) and 15-week-old (15 W) male db/db mice (B6.BKS(D)-Lepr <db>/J) and heterozygous nondiabetic mice (db/m) were purchased from Changzhou Cavens Laboratory Animal Co., Ltd. (Changzhou, China). The mice were maintained in a specific pathogen-free mouse room under a 12-h light/12-h dark cycle. All mouse experimental procedures were performed according to the animal experiment guidelines of the Experimental Animal Management Ordinance of Hubei Province, P. R. China, and the guidelines of Huazhong University of Science and Technology and were approved by the Institutional Animal Ethics Committee of Huazhong University of Science and Technology (IACUC Number: 3139).

### IVCF and *ex vivo* cryofixation (EVCF)

The procedures for IVCF and EVCF are shown in Figure S1. The mice were anesthetized with a mixture of 2% chloral hydrate and 10% ethyl urethane (8 mL/kg body weight, i.p.) and held in a supine position. For IVCF, the abdominal cavity was opened to expose the kidneys, liquid nitrogen was poured over them to achieve a quick freeze, and then the kidneys were removed after approximately half a minute. For EVCF, the kidneys were extracted from anesthetized mice and then frozen on aluminum foil, which floats on liquid nitrogen.

### Micro-CT imaging

We performed *in vivo* imaging in twelve 8 W male C57 mice using a homemade gantry rotation micro-CT system 30. Anesthetized mice were imaged immediately after being injected with the iohexol contrast agent (300 mg iodine/mL, 25 mL/kg body weight, GE, Shanghai, China) through the tail vein. The micro-CT scanner was operated using the following settings: 50 kVp, 800 µA source current, 1 mm aluminum filter, and exposure time of 200 ms, resulting in a matrix size of 1600×1600×1600 with an isotropic voxel size of 39 μm.

After micro-CT imaging, bilateral kidney IVCF and EVCF were performed in six mice each. All of these kidneys were then subjected to cryo-MOST imaging.

### UUO procedure

The UUO operation was performed as previously described 31. A small incision was made on the left flank of 8 W male C57 mice after anesthetization. The left ureter was ligated at two points using 4-0 silk, and the ureter was severed between the two ligations. Then, the muscles and skin were closed in turn. In the control group, ligation was not performed after the abdominal cavity incision. The kidney was subjected to IVCF 3 days after UUO surgery.

### Biochemical characterization and autofluorescence spectra measurements

The mice fasted from 7:00 am to 1:00 pm, and tail venous blood was taken at 1:00 pm. Fasting blood glucose was measured by a glucose meter (ACCU-CHEK^@^ Active, Roche, Germany). Urinary samples were collected from 8:00 a.m. to 10:00 a.m. using metabolic cages. The urinary albumin and creatinine levels were determined using the Albuwell-M Murine microalbuminuria ELISA kit (Exocell, Philadelphia, USA) and the Creatinine Companion kit (Exocell, Philadelphia, USA). Autofluorescence spectra of urine samples were measured by a spectrofluorometer (FP-6500, JASCO, Japan) at room temperature. The wavebands of the excitation and emission spectra were 270-500 nm and 350-650 nm, respectively. Excitation-emission matrix fluorescence spectra were plotted by MATLAB software (2017a, MathWorks).

### Reflux pressure test

The surgical procedures for the reflux pressure tests in the mice were performed as previously described 32, 33. After anesthesia, the abdomens of the mice were surgically opened to expose the urinary bladder and ureters. The adipose tissue around the kidneys and ureters was removed carefully. An IV tube (1 m) was connected to a 26-gauge needle, and a 5 ml syringe filled with methylene blue dye (10 mg/mL) was attached to the opposite end. While holding the bladder firmly with forceps, the 26-gauge needle was inserted into the bladder. A timer set for 30 s was started, and the syringe was raised vertically by 5 cm/min. The hydrostatic pressure (cm-H_2_O) at which urination or reflux occurred was recorded. If reflux did not occur even after urination, the urethral outlet was sealed by a surgical clip. The experiment was then repeated to determine the pressure at which reflux occurred. If vesicoureteral reflux did not occur at 90 cm-H_2_O, the experiment was discontinued, and the “vesicoureteral reflux pressure” was recorded as 90 cm-H_2_O.

### Voiding spot analysis (VSA)

Voiding spot analysis was performed as previously described 34. The bottom of the clean test cages was covered with 16 × 26 cm Whatman Grade 3 filter paper (3030-704, Whatman, UK). Each mouse was placed in a separate test cage without food or water from 9:00 a.m. to 12:00 a.m., allowing the urine to drip onto the filter paper. The filter papers were removed and replaced after each test. The first two days were the adaptive stage for the mice. The filter papers obtained on the third day were imaged under ultraviolet light after testing, showing strong fluorescence from the urine drops. The images were processed using the Void Whizzard plugin 34 of Fiji software (v 2.5.0).

### Image processing and statistical analysis

We manually divided the areas of hydronephrosis and different renal subregions and conducted area statistics using Fiji software. Thirty glomeruli were randomly selected from each kidney for glomerular surface area statistics. One hundred proximal convoluted tubules (PCTs) and proximal straight tubules (PSTs) were randomly selected from each kidney for diameter measurement.

To measure the capillary area fraction, six 200 × 200 pixel^2^ (equal to 330 × 330 μm^2^) raw images were randomly selected in each renal subregion of the cortex, outer stripe of the outer medulla (OSOM), and inner medulla (IM). Raw images in the cortical region were selected to avoid the glomeruli as much as possible. Then, the raw images were sharpened by convolution processing using Fiji software. Next, the capillaries were extracted by threshold processing, and incorrect recognitions were corrected by manual inspection. Then, the fractions of the capillary areas were measured.

3D reconstruction of the kidney was performed by Amira (v 6.1.1, FEI) and Imaris (v 9.0.1, Bitplane AG) software. The volumes of blood vessels were measured using Imaris. Two segmental, eight interlobular, and eight arcuate artery-vein pairs were generated for each kidney using Imaris to calculate their average cross-sectional areas.

We performed the statistical analysis of the data and constructed graphs using GraphPad Prism software (v 7.00, GraphPad). All data are presented as the mean ± SEM. Two-tailed unpaired t tests were used to compare data between different groups. In this study, p < 0.05 was considered significant (* P < 0.05, ** P < 0.01, and *** P < 0.001).

## Results

### Cryo-MOST imaging of normal mouse kidney in 3D

To demonstrate the ability of cryo-MOST to display the 3D anatomical structure of the whole kidney without labeling, we performed autofluorescence cryo-MOST imaging at a resolution of 1.65 × 1.65 × 10 μm of 8 W C57 mouse kidneys (Figure 1A). A total of 528 coronal images of the kidney were acquired, and the dataset size was approximately 29 GB. The 3D kidney reconstruction is shown in Figure 1B. The four main subregions of the kidney, cortex, OSOM, inner stripe of the outer medulla (ISOM), and IM were identified and segmented in 3D and are shown by different colors. In the maximum coronal section of the kidney, we observed that the autofluorescence intensity was the highest in the OSOM but weak in the ISOM and IM (Figure 1C). In contrast, we performed traditional periodic acid-Schiff (PAS) staining and then imaged the results on a similar section of another kidney (Figure 1D). The overall shape of the renal section and the locations of the four subregions acquired by cryo-MOST autofluorescence were consistent with those shown by PAS staining. Typical anatomical structures of the four subregions could be identified in both the cryo-MOST and PAS-stained images, as indicated by yellow squares in Figure 1C-D and as shown enlarged in Figure 1E-L. The blood vessels were black in the cryo-MOST images due to the strong absorption of autofluorescence 35 (Figure 1C, E). The arteries and veins could be distinguished by differences in their contour features and vessel wall thickness (Figure 1E, I, red * and blue *). The arteries were rounder in shape, smaller in diameter, and thicker in the wall than the veins. The glomerular tuft was formed by the lobules of capillaries and appeared as a ball containing dark beads in the cryo-MOST image (Figure 1E, red dotted line). The medullary vascular bundles in the ISOM were also evident in the cryo-MOST image (Figure 1G, blue arrowheads). Benefitting from high contrast, the capillaries were more easily identified and segmented in cryo-MOST sections than in PAS-stained sections (Figure 1E-L, red arrowheads). The boundaries of the renal tubules were clear in cryo-MOST sections (Figure 1E-H). Different segments of renal tubules in different regions showed variations in brightness and morphology in the cryo-MOST images. For example, PCTs in the cortex (Figure 1E, yellow arrows) and PSTs in the OSOM (Figure 1F, blue arrows) showed a larger diameter and higher brightness, while Henle's loop in the ISOM (Figure 1G, white arrows) showed a smaller diameter and lower brightness. These results are morphologically consistent with the observations of the PAS-stained image (Figure 1I-L).

The micron resolution and high contrast of the renal 3D cryo-MOST images allowed us to trace and reconstruct continuous 3D structures *in situ*. We performed a 3D rendering of a 4 × 1 × 1 mm^3^ cuboid from the same dataset as shown in Figure 1B and segmented and reconstructed the renal arteries, veins, glomeruli, and renal tubules (Figure 1M, Supplementary Movie 1). Most veins and arteries were in pairs and close together. The PCT was coiled and extended into the medulla region (Figure 1M, yellow dashed box).

Kidney function depends on the detailed spatial interrelationships among these various microstructures of the kidney. However, due to methodological limitations, it is difficult to quantitatively study the complex 3D anatomy of kidneys, such as the renal vascular system, and its true geometric relationship with specific renal tubule segments 36. Our results demonstrate the ability of cryo-MOST to achieve whole-kidney 3D visualization and allow analysis of multiple renal anatomical structures with cellular resolution. Thus, this method is conducive to the comprehensive assessment of kidney function and observation of renal pathology.

### IVCF preserves the intravital morphology of the whole kidney with little deformation

**Figure 2:**
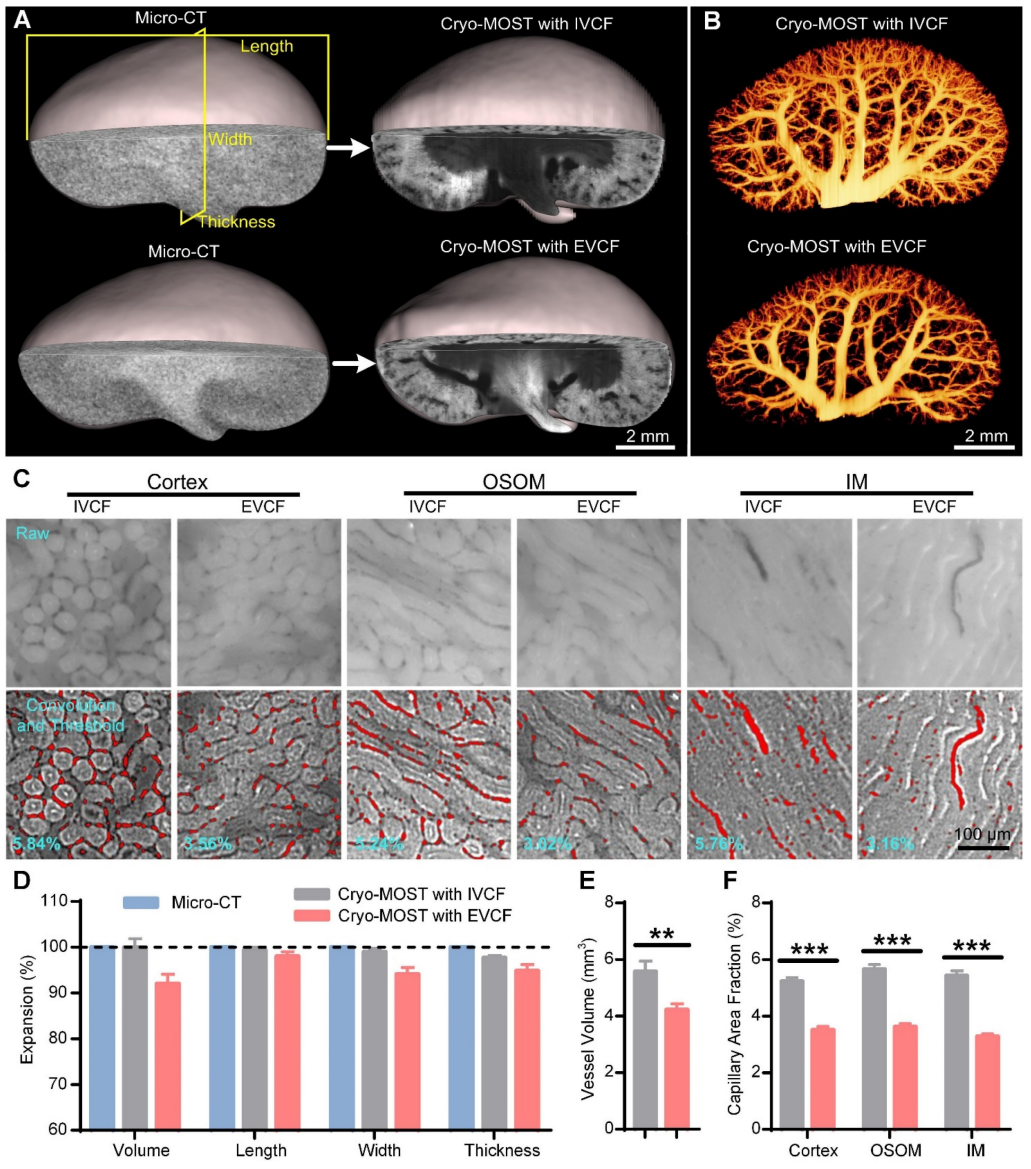
** Evaluation of intravital mouse kidney morphology preservation by *in vivo* cryofixation (IVCF) and *ex vivo* cryofixation (EVCF) for cryo-micro-optical sectioning tomography (cryo-MOST) imaging.** (A) Three-dimensional (3D) reconstruction and cross-sectional display of 3/4 mouse kidneys acquired by cryo-MOST imaging with IVCF and EVCF and the corresponding micro-CT imaging results. (B) Comparison of 3D renal vascular reconstructions obtained by cryo-MOST imaging with IVCF and EVCF. (C) Comparison of capillaries in different subregions of mouse kidneys acquired by cryo-MOST imaging with IVCF and EVCF. The red marks indicate segmented capillaries. (D) Quantification of volumetric and linear expansion of whole kidneys acquired by cryo-MOST imaging with IVCF and EVCF compared with micro-CT imaging results. (E) Vessel volumes of the kidneys. (F) Capillary area fractions in different renal subregions. N = 12 kidneys for each group. ** P < 0.01, *** P < 0.001.

The traditional cryofixation method for kidneys is to remove the kidney from the body and then freeze it. However, the blood and fluid in the renal pelvis flow out when the kidney is isolated, which changes the perfusion status as well as the overall shape of the kidney. IVCF of the kidneys with liquid nitrogen while mice are alive may be promising to maintain the original morphology and perfusion status of the kidney. Thus, we further evaluated the effect of IVCF and EVCF on preserving the obtaine of intravital mouse kidneys by cryo-MOST imaging. The original morphology of the kidneys was obtained by intravital imaging using micro-CT as a control. We performed 3D reconstruction and generated three orthogonal sectional images of the acquired micro-CT and cryo-MOST images (Figure 2A, Figure S2, Figure S3). The 3D and sectional morphology of the kidney as observed by cryo-MOST with IVCF was similar to that of the kidney as observed by micro-CT (Figure S2), while the kidney observed by cryo-MOST with EVCF showed partial deformation (Figure S3B blue arrow). The deformation of the kidney by EVCF may be because the kidney was compressed during removal or freezing, which is difficult to avoid completely. Then, the volumetric and linear expansion of whole kidneys were further analyzed statistically, as shown in Figure S2 and Figure S3. We found little change in the kidney volume, length, width, and thickness between cryo-MOST with IVCF and micro-CT, with expansion percentages of 99.95 ± 1.92%, 98.84 ± 0.68%, 98.37 ± 0.91%, and 97.15 ± 0.66%, respectively (Figure 2D). In contrast, the corresponding parameters for cryo-MOST with EVCF were 92.0 ± 2.08%, 98.01 ± 1.00%, 94.07 ± 1.49%, and 94.82 ± 1.36% (Figure 2D). These results indicate that IVCF preserved the *in vivo* morphology of the kidneys with less deformation, while the excised kidneys shrank, possibly due to blood outflow.

Small changes in vascular morphology can have a disproportionately large effect on estimated transport rates 37, so accurate acquisition of vascular morphology in the fluid perfusion status is crucial to computational modeling of renal oxygenation. Using semiautomatic threshold segmentation, we reconstructed and compared the 3D blood vessels in cryo-MOST images of kidneys obtained by IVCF and EVCF (Figure 2B). The density of the renal blood vessels in the EVCF group was lower than that in the IVCF group, especially the small blood vessels (Figure 2B). The volume of blood vessels in the EVCF group was reduced by 24.18% compared with that in the IVCF group (Figure 2E). We also measured the area fractions of capillaries in the cortex, OSOM, and IM (Figure 2C) and found that those of kidneys obtained by cryo-MOST with EVCF were reduced by 32.81%, 35.89%, and 39.51% compared with those obtained by cryo-MOST with IVCF, respectively (Figure 2F). These results indicate that IVCF better preserved the anatomical morphology of blood vessels in the kidney than EVCF. IVCF combined with cryo-MOST potentially provides valuable information for studying the process of blood oxygen transport, filtration, and reabsorption.

### 3D cryo-MOST imaging of UUO mouse kidneys

**Figure 3:**
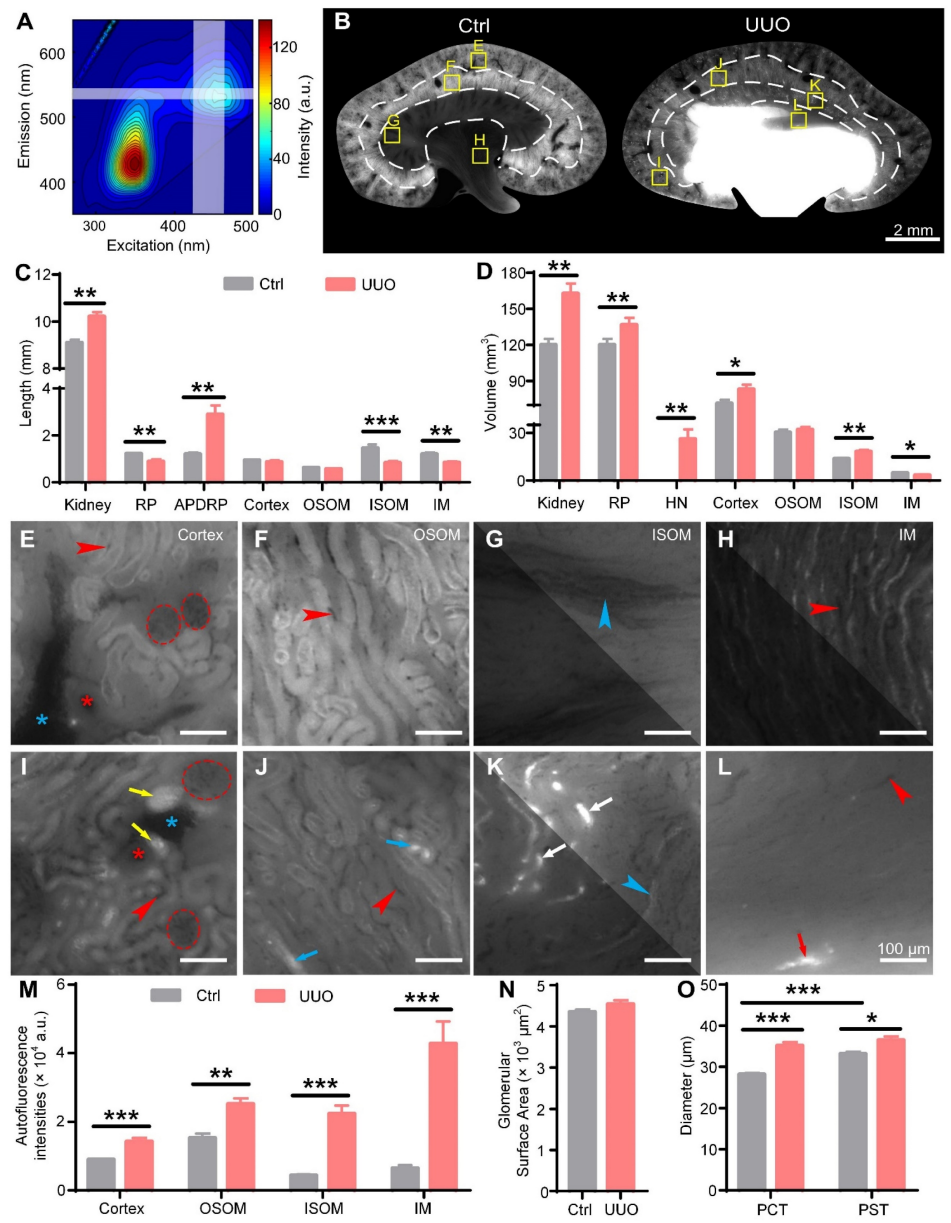
** Cryo-micro-optical sectioning tomography (cryo-MOST) imaging of control and unilateral ureteral obstruction (UUO) mouse kidneys.** (A) Excitation-emission matrix fluorescence spectra of C57 mouse urine. The white translucent band indicates the working wavelength of the cryo-MOST system. (B) Typical coronal images of control and 3 days post-UUO mouse kidneys acquired by cryo-MOST. The white dotted lines indicate the boundaries of the renal subregions. The exposure times for imaging the control and UUO kidneys were 200 ms and 100 ms, respectively. (C-D) Lengths and volumes of whole kidneys and renal subregions in the control and UUO mice. RP, renal parenchyma; APDRP, anteroposterior diameter of the renal pelvis; OSOM, outer stripe of the outer medulla; ISOM, inner stripe of the outer medulla; IM, inner medulla; HN, hydronephrosis. (E-L) Enlarged views of the corresponding yellow rectangles in (B). We adjusted the contrast in the upper right parts of (G, H, and K) to facilitate viewing. Some proximal convoluted tubules (PCTs, yellow arrows), proximal straight tubules (PSTs, blue arrows), Henle's loops (white arrows), and collecting ducts (red arrows) were dilated in the UUO mouse kidneys. Red dotted line, glomeruli; red *, arteries; blue *, veins; blue arrowheads, medullary vascular bundles; red arrowheads, capillaries. (M) Autofluorescence intensities of the renal subregions in the control and UUO mouse kidneys. (N) Glomerular surface area. (O) Diameters of PCTs and PSTs. Control N = 6, UUO N = 5. * P < 0.05, ** P < 0.01, *** P < 0.001.

To evaluate the feasibility of cryo-MOST to observe hydronephrosis, we measured the excitation-emission matrix fluorescence spectra of mouse urine (Figure 3A) and found that there were two distinct fluorescence emission peaks, consistent with previous studies 38, 39. The emission peak at approximately 430 nm (excitation: 350 nm) may be mainly ascribed to the presence of nicotinamide adenine dinucleotide (NADH) and nicotinamide adenine dinucleotide phosphate (NADPH), and the other emission peak at approximately 530 nm (excitation: 450 nm) may be attributed to riboflavin and its metabolic derivatives, such as flavin adenine dinucleotide (FAD) and flavin mononucleotide 38, 39. The imaging band of the cryo-MOST system coincided with the latter fluorescence peak (Figure 3A, white translucent band), which was convenient for us to obtain the distribution of hydronephrosis with autofluorescence. Next, we performed cryo-MOST imaging to observe hydronephrosis and the resultant renal parenchymal lesions in 3 days after UUO mice kidneys. We found that the autofluorescence intensity of the UUO mouse kidneys was higher than that of the control kidneys and then set the imaging exposure time for the UUO and control mouse kidneys at 100 and 200 ms, respectively.

We performed 3D renderings of UUO and control mouse kidneys and then generated three orthogonal sectional images. The UUO kidneys were larger than the control kidneys in the 3D renderings (Figure S4A). The renal pelvis in the UUO mouse kidneys was highlighted, indicating hydronephrosis, which had expanded into the ISOM and caused serious deformation of the renal parenchyma (Figure 3B, Figure S4A-C). The control kidneys showed a uniform tissue distribution without hydronephrosis (Figure 3B, Figure S4B-C). The autofluorescence intensities of the ISOM and IM were much lower than those of the OSOM in the control kidneys. However, in UUO kidneys, the autofluorescence intensities of the ISOM and IM were similar to those of the OSOM (Figure 3B). By further statistical analysis, we found that the autofluorescence intensities of each renal subregion in the UUO kidneys were significantly higher than those of the controls, especially that of the ISOM (Control: 4374 ± 278 a.u., UUO: 22363 ± 2383 a.u.) and IM (Control: 6450 ± 861 a.u., UUO: 42712 ± 6512 a.u.) (Figure 3M), possibly due to crude urine accumulation in the dilated renal tubules.

The evaluation of hydronephrosis is of great reference value for the selection of treatment. Traditional methods for grading hydronephrosis mainly measure the kidney length, anteroposterior diameter of the renal pelvis (APDRP), and renal parenchyma thickness or observe the distribution of hydronephrosis through 2D imaging 40. We measured these length parameters in the orthogonal sectional images of the control and UUO kidneys accordingly (Figure S4B-C). The kidney length (Control: 9.10 ± 0.12 mm, UUO: 10.21 ± 0.20 mm) and APDRP (Control: 1.20 ± 0.06 mm, UUO: 2.89 ± 0.38 mm) were increased in the UUO kidneys compared with the control kidneys, while the UUO kidneys showed a significantly decreased renal parenchyma thickness (Control: 3.01 ± 0.05 mm, UUO: 2.30 ± 0.14 mm) (Figure 3C). These thickness measurements are consistent with previous studies and are considered evidence of renal parenchymal atrophy 9, 40. We also measured the thickness of the four main subregions of the kidneys. There was no significant difference in the cortex (Control: 0.94 ± 0.03 mm, UUO: 0.87 ± 0.06 mm) or OSOM (Control: 0.62 ± 0.03 mm, UUO: 0.56 ± 0.03 mm) between the control and UUO kidneys, while the thickness of the ISOM (Control: 1.46 ± 0.04 mm, UUO: 0.83 ± 0.08 mm) and IM (Control: 1.20 ± 0.06 mm, UUO: 0.84 ± 0.05 mm) was decreased significantly in the UUO kidneys (Figure 3C).

3D imaging allowed us to further measure the volumes of these renal regions. We found that the kidney volumes in the UUO mice were significantly increased compared with those in the control mice (Control: 120 ± 4.96 mm^3^, UUO: 162.80 ± 8.40 mm^3^) (Figure 3D). The volumes of hydronephrosis reached 26.11 ± 6.00 mm^3^ in the UUO kidneys, while no hydronephrosis was observed in the control kidneys (Figure 3D). The volume of the renal parenchyma in the UUO kidneys was higher than that in the control kidneys (Control: 120 ± 4.96 mm^3^, UUO: 136.7 ± 5.84 mm^3^), although the difference was not statistically significant (Figure 3D). There was no significant difference in the OSOM volume between the UUO (32.08 ± 1.65 mm^3^) and control (30.21 ± 1.83 mm^3^) kidneys, while there was a decrease in the IM volume (Control: 4.82 ± 0.34 mm^3^, UUO: 3.34 ± 0.44 mm^3^) in the UUO kidneys (Figure 3D). The volumes of the cortex (Control: 71.30 ± 3.00 mm^3^, UUO: 83.05 ± 3.83 mm^3^) and ISOM (Control: 13.70 ± 0.45 mm^3^, UUO: 18.18 ± 1.13 mm^3^) in the UUO kidneys were significantly higher than those in the control kidneys (Figure 3D). The thickness measurements of the OSOM and IM were consistent with the volume measurements, indicating that the OSOM was unchanged while the IM was atrophic. However, while the thickness of the renal parenchyma and ISOM showed a decrease, the volume of these regions showed an increase, indicating dilation of these regions rather than atrophy in the early stage of UUO. Thus, traditional length measurements in 2D section images cannot accurately reflect 3D volume changes. The different changes in the renal subregions of UUO mice indicated that the injury mechanisms may be different. Therefore, attention should be given to distinguishing different subregions when studying the injury process and treatment of UUO kidneys.

Then, we observed the morphology of the nephrons in the control and UUO kidneys (Figure 3E-L). The glomeruli showed no significant morphological change between the control and UUO kidneys (Figure 3E, I), and the statistical results of the glomerular surface area (Control: 4352 ± 57 μm^2^, UUO: 4539 ± 96 μm^2^) also confirmed this observation (Figure 3N). However, many PCTs (Figure 3L, yellow arrows) in the cortex and Henle's loops (Figure 3K, white arrows) in the ISOM were centrally highlighted and bulged in the UUO kidneys. This result indicated that the renal tubules were dilated due to the extrusion of crude urine. Only a small number of PSTs (Figure 3J, blue arrows) in the OSOM and collecting ducts (Figure 3L, red arrows) in the IM were dilated in the UUO kidneys. This is consistent with the increase in the volume of the cortex and ISOM in the UUO kidneys mentioned above (Figure 3D). By further analysis of the proximal tubules, we found that the diameter of the PSTs was significantly larger than that of the PCTs in the control kidneys, but there was no significant difference between the PSTs and PCTs in the UUO kidneys. Compared with the control kidneys, the diameters of the PCTs (Control: 28.13 ± 0.34 μm, UUO: 35.13 ± 0.87 μm) and PSTs (Control: 33.11 ± 0.55 μm, UUO: 36.48 ± 0.91 μm) in the UUO kidneys were increased by 24.9% and 10.2%, respectively (Figure 3O). These data indicate that PCTs and Henle's loops are more susceptible to hydronephrosis.

**Figure 4:**
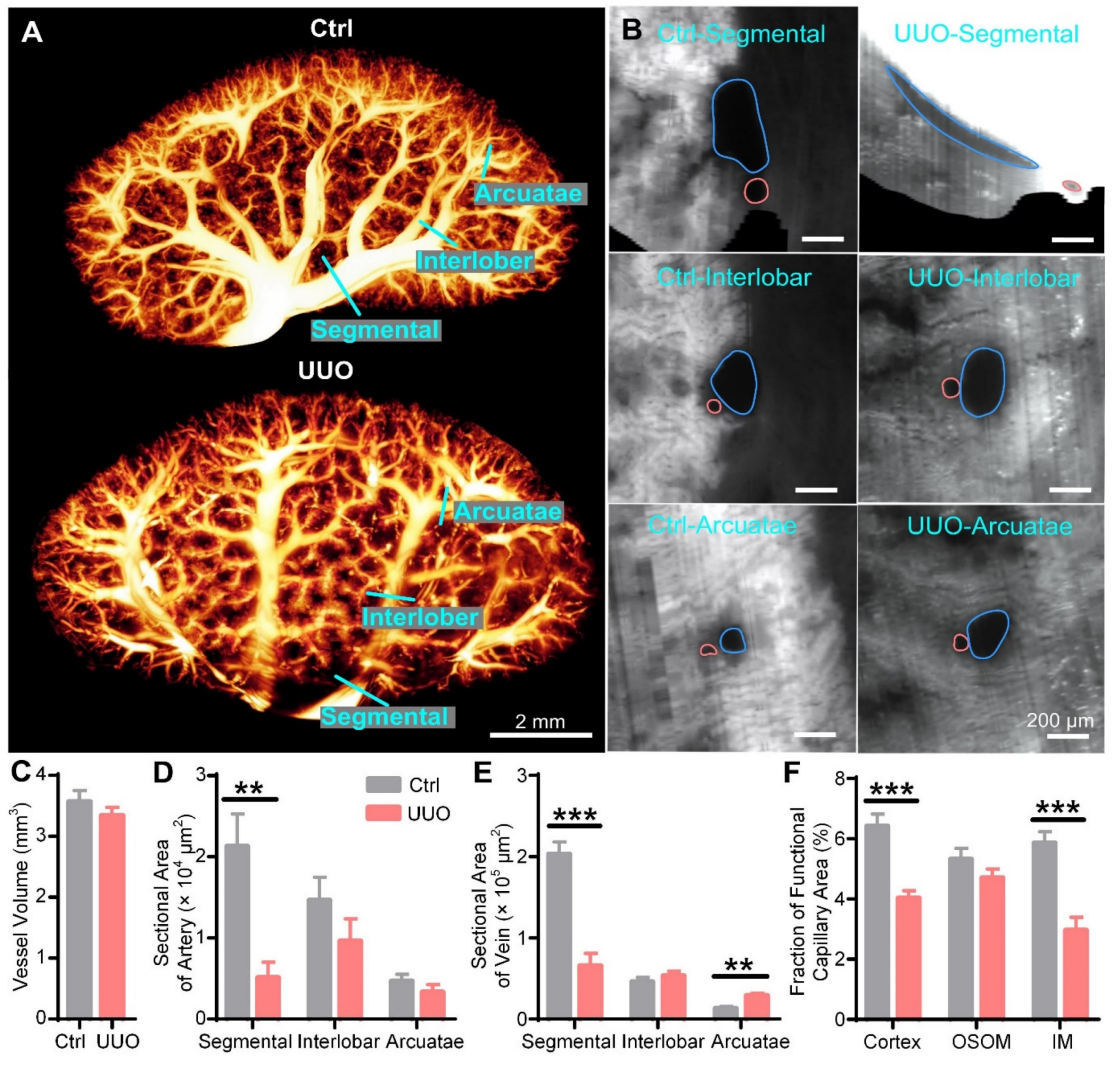
** Three-dimensional (3D) reconstruction and quantification of renal vessels in control and unilateral ureteral obstruction (UUO) mouse kidneys.** (A) 3D reconstruction of renal vessels. (B) Cross-sections of different hierarchical levels of artery (red lines) and vein (blue lines) pairs indicated by cyan lines in (A). (C) Vessel volumes in the control and UUO mouse kidneys. (D- E) Cross-sectional areas of different hierarchical levels of arteries (D) and veins (E). (F) Fractions of the functional capillary areas in different renal subregions. Control N = 6, UUO N = 5. ** P < 0.01, *** P < 0.001.

We also reconstructed and quantified the blood vessels in UUO and control kidneys in 3D, with the smallest vessel being approximately 15 μm in diameter. The blood vessels in the control kidneys showed numerous branches from the hilar region to the periphery, and most of them decreased in diameter. However, the segmental vessels near the renal hilum in the UUO kidneys were significantly atrophied, but the interlobar and arcuate vessels were full (Figure 4A, Supplementary Movie 2). To further evaluate the vascular changes, we generated cross-sections of the segmental, interlobar, and arcuate arteries and veins (Figure 4B). The segmental arteries and veins of the UUO kidneys were elongated, while those of the control kidneys were thick and round. There was no significant difference in the shape of arcuate or interlobular vessels between the UUO and control kidneys (Figure 4B). To quantify the renal vascular changes, we measured the volumes of blood vessels and calculated the cross-sectional areas of the arteries and veins. We found no significant difference in blood vessel volume between the UUO and control kidneys (Control: 3.57 ± 0.18 mm^3^, UUO: 3.34 ± 0.13 mm^3^) (Figure 4C). Compared to the control kidneys, the cross-sectional area of segmental arteries (Control: 21281 ± 3979 μm^2^, UUO: 5116 ± 1877 μm^2^) was significantly decreased in the UUO kidneys, while those of interlobar arteries (Control: 14628 ± 2830 μm^2^, UUO: 9625 ± 2718 μm^2^) and arcuate arteries (Control: 4699 ± 820 μm^2^, UUO: 3308 ± 919 μm^2^) were also decreased, although the difference was not statistically significant (Figure 4D). Regarding the veins, compared to the control kidneys, the UUO kidneys showed a decrease in the cross-sectional area of segmental veins (Control: 203284 ± 14904 μm^2^, UUO: 65773 ± 15228 μm^2^), no difference in that of interlobar veins (Control: 46043 ± 5256 μm^2^, UUO: 53243 ± 5822 μm^2^), and an increase in that of arcuate veins (Control: 13259 ± 2019 μm^2^, UUO: 28954 ± 2704 μm^2^) (Figure 4E). The decrease in the cross-sectional area of the segmental arteries and veins in the UUO kidneys may be caused by the compression of accumulated hydronephrosis, which may contribute to the previously reported poor blood flow and increased vascular pressure in UUO kidneys 41, 42. The increased cross-sectional area of arcuate veins may also be caused by increased vascular pressure.

Impaired blood perfusion is considered an important cause of renal hypoxia and fibrosis in UUO mice 18, 43, 44. Functional capillaries can directly reflect the conditions of blood perfusion *in vivo* and are sensitive and objective indicators for evaluating renal ischemia and hypoxia. However, previous studies only observed the functional capillary status of the local renal cortex via two-photon microscopy 45, 46. Current 3D whole-kidney imaging usually requires dehydration and fixation, resulting in loss of functional information regarding perfusion. Therefore, the functional capillaries of the whole kidney have not been effectively assessed. Here, we evaluated the functional capillary density in the cortex, OSOM, and IM of the control and UUO kidneys (Figure S5). Compared with the control kidneys, the functional capillary area fractions in the cortex (Control: 6.42 ± 0.40%, UUO: 4.03 ± 0.25%) and IM (Control: 5.86 ± 0.37%, UUO: 2.96 ± 0.43%) of the UUO kidneys were reduced by 37.2% and 49.5%, respectively (Figure 4F). However, there was no significant difference in that of the OSOM (Control: 5.31 ± 0.37%, UUO: 4.70 ± 0.30%) between the two groups (Figure 4F). This result indicated that the microcirculation of the renal cortex and IM in UUO mice was impaired and that hypoxia might have occurred in these regions, while the OSOM was normal. Interestingly, as shown in Figure 3, the cortex and IM were more susceptible to hydronephrosis than the OSOM, which may be related to functional capillary rarefaction in the cortex and IM rather than in the OSOM. Therefore, cryo-MOST imaging can provide unique insights into the diagnosis of early renal lesions and enable exploration of the role of ischemia and hypoxia in renal disease development.

### 3D cryo-MOST imaging of db/db mouse kidneys

The db/db mouse is a commonly used model of type 2 diabetes and exhibits spontaneous hydronephrosis 47, but its etiology, progression, and effects are still unknown. By cryo-MOST imaging, we found that all db/db mice had mild hydronephrosis at 6 W (Figure S6A, E), which is considered the time when blood glucose begins to increase 48. Our results also showed that the blood glucose of 6 W db/db mice was slightly higher than that of 6 W db/m mice, but the difference was not statistically significant (Figure S6B). The body weight of 6 W db/db mice was already significantly higher than that of db/m mice (Figure S6C), but there was no difference in kidney volume (Figure S6D). These results suggest that hydronephrosis may appear earlier than diabetes in db/db mice and cause long-term kidney damage. Therefore, it is necessary to study the effect of hydronephrosis on the kidneys of db/db mice. Previous studies have shown that db/db mice have high blood glucose levels at 15 W and develop some typical symptoms of diabetes 48. Therefore, we selected 15 W db/db mice and normal db/m mice for further study. The blood glucose (db/m: 24.23 ± 1.071 mmol/L, db/db: 6.61 ± 0.37 mmol/L), body weight (db/m: 52.8 ± 1.09 g, db/db: 33.47 ± 0.77 g), kidney weight (db/m: 0.215 ± 0.005 g, db/db: 0.181 ± 0.006 g) and albumin-to-creatinine ratio (ACR, db/m: 554.80 ± 73.51 μg/mg·Cr, db/db: 58.38 ± 19.06 μg/mg·Cr) of 15 W db/db mice were significantly higher than those of db/m mice (Figure S7), consistent with previous studies 48, confirming that the 15 W db/db mice showed obvious symptoms of diabetes.

**Figure 5:**
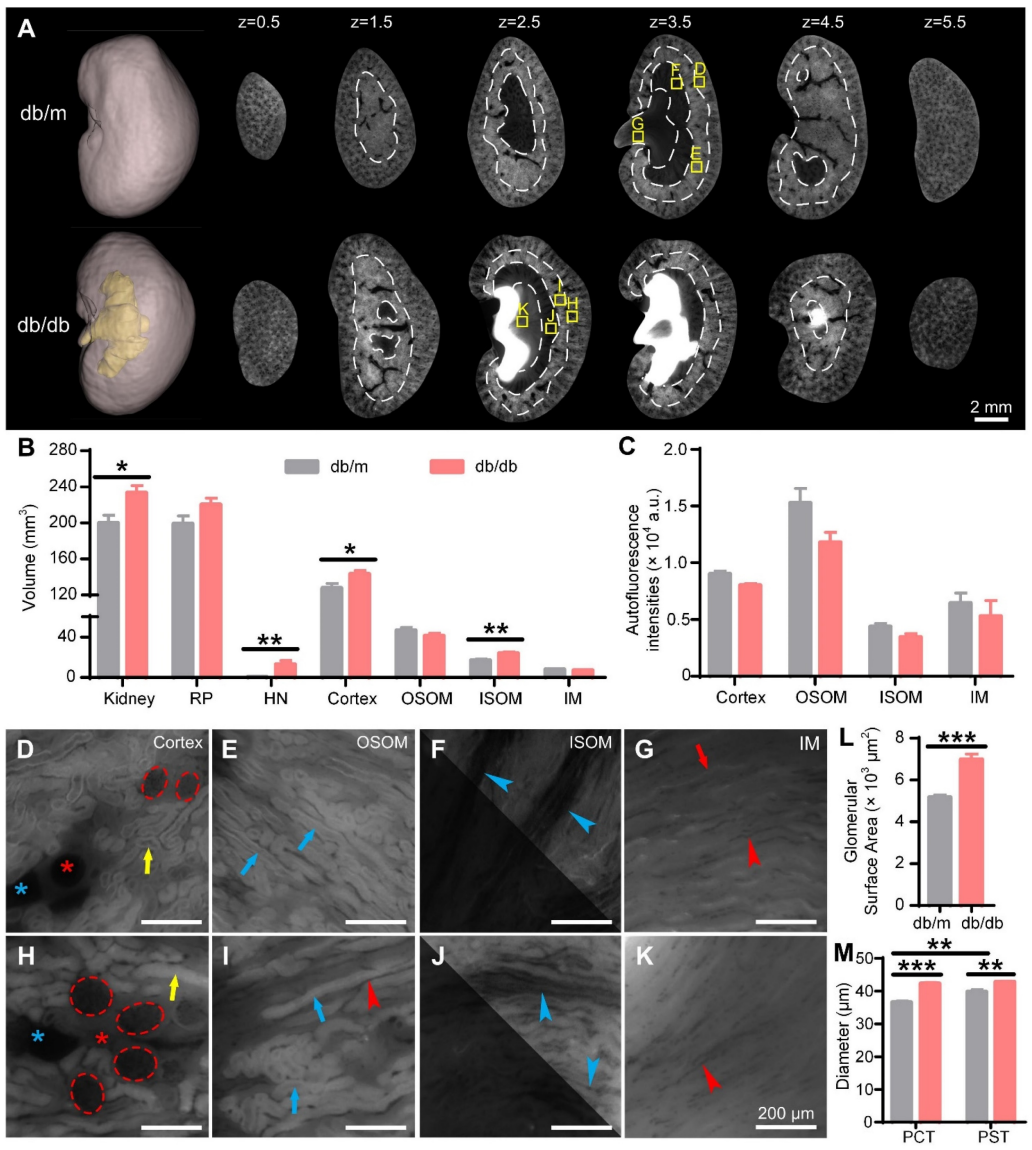
** Cryo-micro-optical sectioning tomography (cryo-MOST) imaging of 15-week-old db/m and db/db mouse kidneys.** (A) Three-dimensional (3D) reconstruction and equally spaced sequential section images of db/m and db/db mouse kidneys. The yellow fill in the 3D reconstruction represents hydronephrosis. The white dotted lines indicate the boundaries of the renal subregions. (B) Volumes of the whole kidney and renal subregions in db/m and db/db mice. RP, renal parenchyma; HN, hydronephrosis; OSOM, outer stripe of the outer medulla; ISOM, inner stripe of the outer medulla; IM, inner medulla. (C) Autofluorescence intensities of renal subregions in the db/m and db/db mouse kidneys. (D-K) Enlarged views of the corresponding yellow squares in (A). We adjusted the contrast in the upper right parts of (F, J) to facilitate viewing. Red dotted lines, glomeruli; red *, arteries; blue *, veins; yellow arrows, proximal convoluted tubules (PCTs); blue arrows, proximal straight tubules (PSTs); red arrows, collecting ducts; blue arrowheads, medullary vascular bundles; red arrowheads, capillaries. (L) Glomerular surface area. (M) Diameters of PCTs and PSTs. Db/m N = 6, db/db N = 5. * P < 0.05, ** P < 0.01, *** P < 0.001.

Consistent with C67 mouse kidneys, the four main subregions of the 15 W db/db and db/m mouse kidneys were identified in cryo-MOST sequence images (Figure 5A). Unlike the UUO model, the autofluorescence intensities of the four renal subregions in the 15 W db/db mice were not significantly different from those in the db/m mice (Figure 5A, C). The whole kidney volume (db/m: 199.7 ± 8.75 mm^3^, db/db: 233.20 ± 8.16 mm^3^) of 15 W db/db mice was larger than that of db/m mice (Figure 5B). Hydronephrosis invaded the ISOM in all 15 W db/db mice (Figure 5A). The average volume of hydronephrosis reached 13.01 ± 3.74 mm^3^ in 15 W db/db mice, and the majority of db/m mice had no hydronephrosis (only one db/m mouse had mild hydronephrosis) (Figure 5B). The volume of the renal parenchyma (db/m: 199.00 ± 8.67 mm^3^, db/db: 220.20 ± 7.26 mm^3^) in the 15 W db/db mice was higher than that in the db/m mice, but the difference was not statistically significant (Figure 5B). Therefore, the renal enlargement in the 15 W db/db mice observed in previous studies 48 may be partly due to the increase in hydronephrosis. Regarding renal subregions, the volume of the cortex (db/m: 127.60 ± 5.15 mm^3^, db/db: 143.00 ± 3.88 mm^3^) and ISOM (db/m: 16.90 ± 1.33 mm^3^, db/db: 23.91 ± 1.41 mm^3^) was higher in 15 W db/db mouse kidneys than in db/m mouse kidneys, while there was no significant difference in that of the OSOM (db/m: 46.60 ± 2.88 mm^3^, db/db: 41.06 ± 2.59 mm^3^) or IM (db/m: 7.95 ± 0.55 mm^3^, db/db: 6.95 ± 0.29 mm^3^) (Figure 5B). The change in the volume of the cortex and ISOM in the 15 W db/db mouse kidneys was the same as that in the UUO model (Figure 3D, Figure 5E). These data suggest that the development of hydronephrosis may contribute to the changes in the renal parenchyma, cortex, and ISOM volume, which merits attention in diabetic nephropathy studies using db/db mice.

We further evaluated the morphology of the nephrons in 15 W db/db and db/m mouse kidneys (Figure 5D-K). The glomeruli of 15 W db/db kidneys were significantly larger than those of db/m kidneys (Figure 5D, H), and the statistical results of the glomerular surface area (db/m: 5170 ± 116 μm^3^, db/db: 6976 ± 263 μm^3^) confirmed this observation (Figure 5L), consistent with previous studies 48, 49. Compared with 15 W db/m mouse kidneys, the PCT (db/m: 36.57 ± 0.43 μm, db/db: 42.29 ± 0.35 μm) and PST (db/m: 39.80 ± 0.69 μm, db/db: 42.80 ± 0.29 μm) diameters in the db/db mouse kidneys were increased by 15.6% and 7.5%, respectively (Figure 5M). The diameter of PSTs was significantly larger than that of PCTs in the 15 W db/m mouse kidneys, but there was no significant difference in the db/db mouse kidneys. Renal tubule dilatation was observed in the kidneys of both 15 W db/db and 3 days post-UUO mice, which was consistent with previous studies using stained sections 50. However, unlike the UUO model, the renal tubules of 15 W db/db mice showed a normal morphology and no central bulge highlighting (Figure 3I-L, Figure 5H-K). This may result from differences in the etiology and severity of hydronephrosis between the two models. These results indicate that cryo-MOST can be used to not only effectively display and analyze spontaneous hydronephrosis in db/db mice but also distinguish the different types of structural damage caused by chronic and acute hydronephrosis.

**Figure 6:**
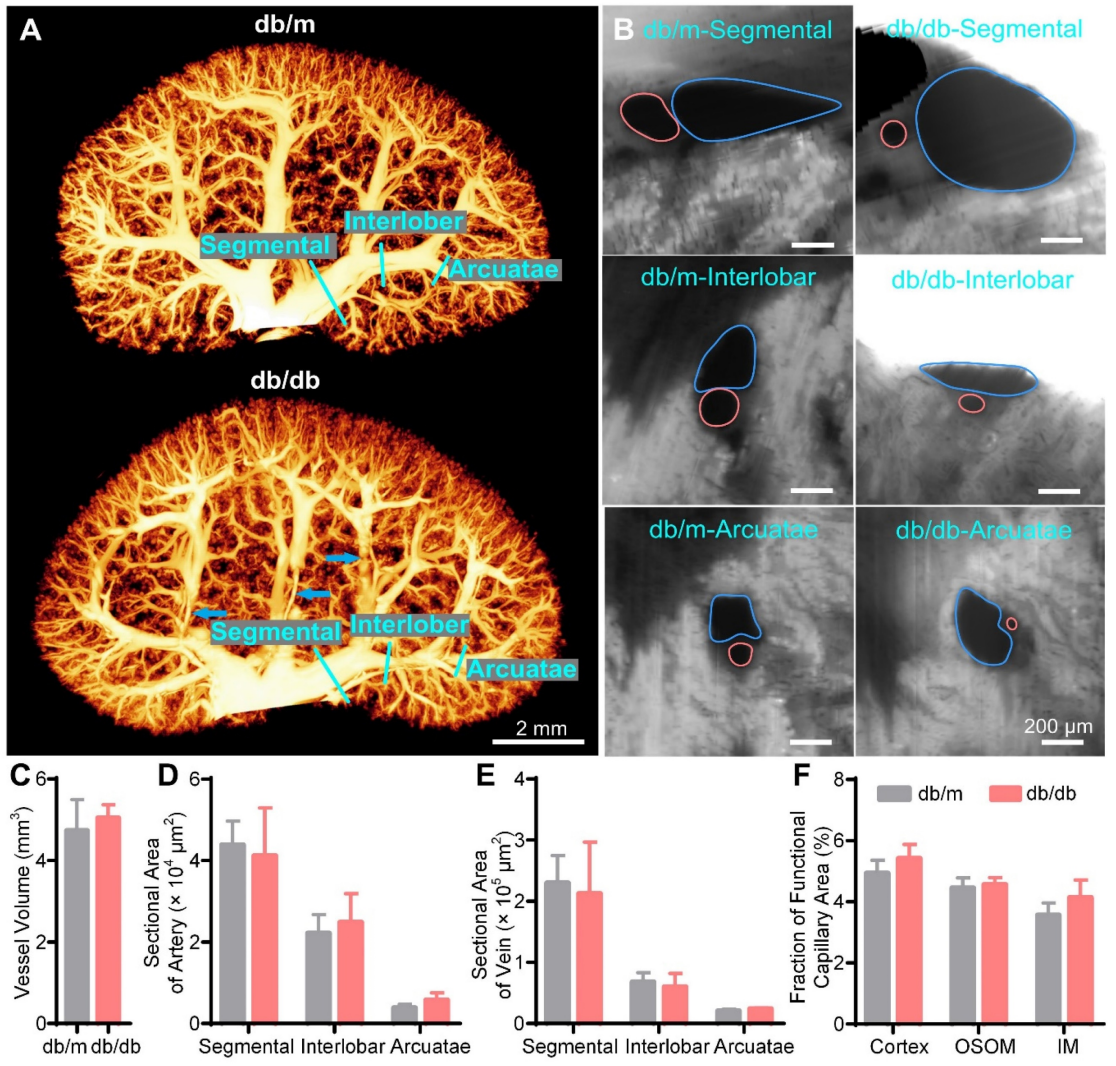
** Three-dimensional (3D) reconstruction and quantification of renal vessels in 15-week-old db/m and db/db mouse kidneys.** (A) 3D reconstruction of the renal vessels in db/m and db/db mouse kidneys. The blue arrows indicate the deformation of some interlobar veins by hydronephrotic compression. (B) Cross-sections of different hierarchical levels of artery (red lines) and vein (blue lines) pairs indicated by cyan lines in (A). (C) Vessel volumes in db/m and db/db mouse kidneys. (D-E) Cross-sectional areas of different hierarchical levels of arteries (D) and veins (E). (F) Fractions of the functional capillary area in different renal subregions. Db/m N = 6, db/db N = 5.

Then, we reconstructed and quantified the blood vessel network in the 15 W db/db and db/m mouse kidneys. There was no significant difference in the overall morphology of renal vessels between the 15 W db/db and db/m mouse kidneys (Figure 6A, Supplementary Movie 3); only some interlobar vessels were deformed (Figure 6A, blue arrows). Additionally, there was no significant difference in the vessel volume between the 15 W db/db and db/m mouse kidneys (db/m: 4.73 ± 0.76 mm^3^, db/db: 4.95 ± 0.40 mm^3^) (Figure 6C). In cross-sectional images of the hierarchical order of artermgies and veins, we found that there was no significant difference in the morphology or size of most blood vessels between 15 W db/db and db/m mouse kidneys, and only some of the interlobar veins adjacent to areas of hydronephrosis were oblate (Figure 6B). Statistical results of the cross-sectional areas of different hierarchical levels of veins and arteries showed no significant differences between 15 W db/m and db/db mouse kidneys (Figure 6D, E). This difference from the acute obstruction hydronephrosis model may result from the fact that the severity of hydronephrosis in the 15 W db/db mice is not as serious as that in the 3 days post-UUO mice (hydronephrosis volume, db/db: 13.01 ± 3.74 mm^3^, UUO: 26.11 ± 6.00 mm^3^). These results suggest that the progression of hydronephrosis had a slight effect on renal vascular morphology in 15 W db/db mice kidneys. However, it should be noted that the severity of hydronephrosis in the db/db mice increased with age, and we cannot rule out the possibility that hydronephrosis has more adverse effects on kidneys with increasing age.

We also measured the peritubular functional capillary density in the 15 W db/db and db/m mouse kidneys (Figure S8). In contrast to the decreased functional capillary area fraction in the 3 days post-UUO mice, we found that there was no difference in the functional capillary area fraction of the three renal subregions between db/db and db/m mice (Figure 6F). This indicates that the peritubular functional capillaries in the db/db mice do not exhibit quantitative abnormalities.

### Vesicoureteral reflux and bladder overactivity in db/db mice

**Figure 7:**
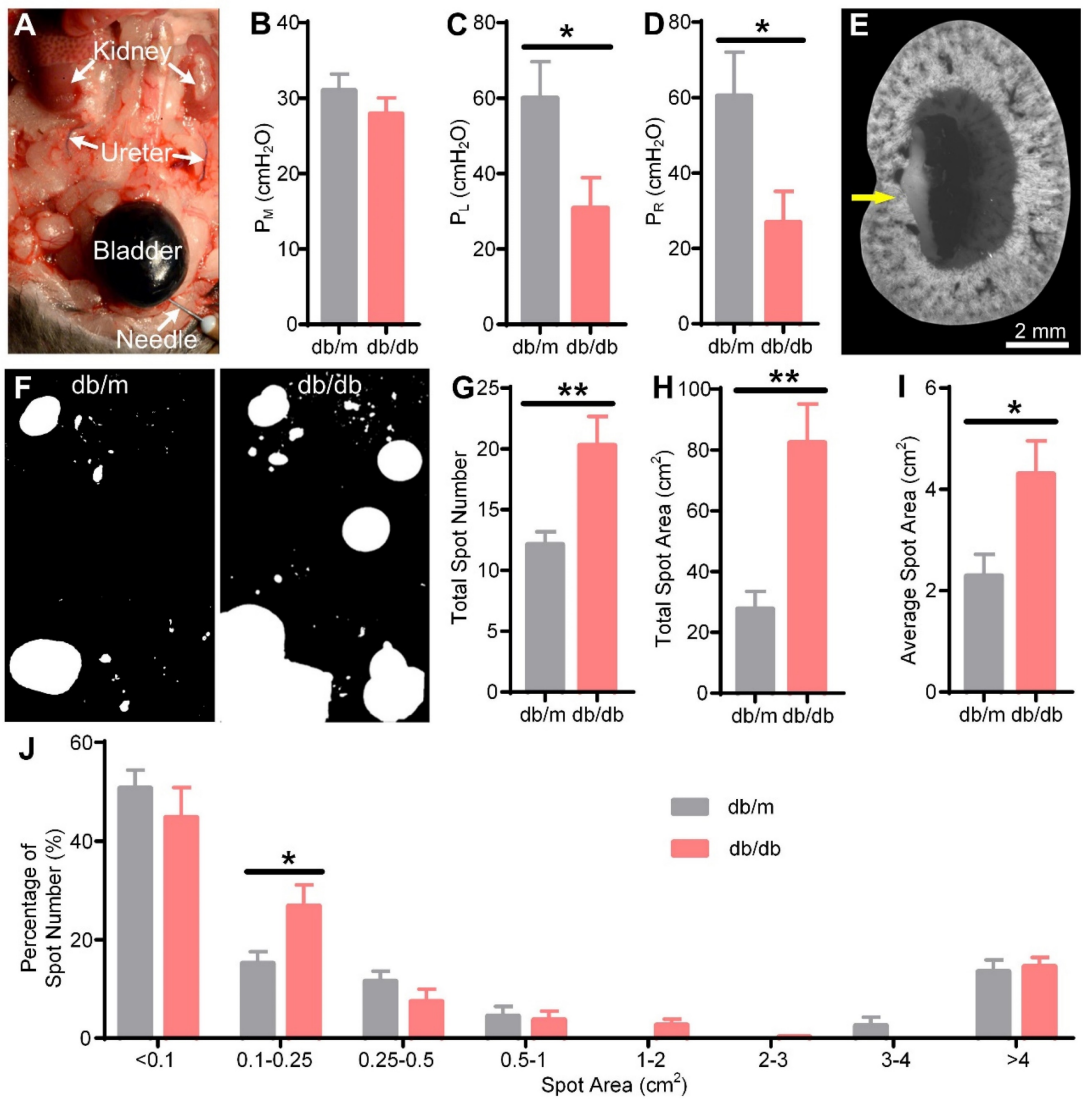
** Vesicoureteral reflux and bladder overactivity in 15-week-old db/db mice.** (A) Reflux pressure was determined by injecting methylene blue solution into the bladder. The arrows indicate methylene blue solution reflux into the ureter from the bladder in the db/db mice. (B) Micturition pressure (P_M_) of db/m and db/db mice. (C) Left reflux pressure (P_L_). (D) Right reflux pressure (P_R_). (E) The db/db mouse kidney was imaged by cryo-micro-optical sectioning tomography after the reflux pressure test. The yellow arrow indicates methylene blue solution reflux into the kidney. (F) Representation of urine spots in the db/m and db/db mice. The filter paper was 16 × 26 cm. (G) Total spot number. (H) Total spot area. (I) Average spot area. (J) Frequency distribution of urine spots of different sizes. Db/m N = 8, db/db N = 9. * P < 0.05. ** P < 0.01.

We next explored the cause of hydronephrosis in db/db mice. Common causes of hydronephrosis are upper or lower urethral obstruction, vesicoureteral reflux, and bladder dysfunction, among others 1-3. We observed smooth flow from the ureter to the bladder by injecting methylene blue solution into the kidney in 15 W db/db mice (data not shown), so we excluded ureteral obstruction as a cause. Arthur A. Like et al. also reported that no ureteral abnormalities were found in db/db mice 47. Then, we performed a reflux pressure test in 15 W db/db and db/m mice (Figure 7A). We found that there was no significant difference in the micturition pressure (db/m: 31.00 ± 2.20 cmH_2_O, db/db: 27.89 ± 2.13 cmH_2_O) between db/db and db/m mice (Figure 7B), indicating no obstruction of the lower urethra in the db/db mice. However, both the left (db/m: 60.00 ± 9.70 cmH_2_O, db/db: 30.78 ± 8.14 cmH_2_O) and right (db/m: 60.38 ± 11.69 cmH_2_O, db/db: 26.89 ± 8.24 cmH_2_O) ureteral reflux pressures in the db/db mice were significantly lower than those in the db/m mice (Figure 7C-D), indicating that the db/db mice had bilateral vesicoureteral reflux. Then, we harvested the kidneys by IVCF and performed cryo-MOST imaging. We found that methylene blue had refluxed into the renal medulla in the db/db mice (Figure 7E), confirming the presence of vesicoureteral reflux.

Furthermore, we evaluated the presence of bladder abnormalities in the db/db mice through the VSA experiment (Figure 7F). The spot number (db/m: 12.13 ± 1.06, db/db: 20.27 ± 2.38), total spot area (db/m: 27.67 ± 5.78 cm^2^, db/db: 82.36 ± 12.76 cm^2^), and average spot area (db/m: 2.29 ± 0.43 cm^2^, db/db: 4.30 ± 0.66 cm^2^) of db/db mice were increased compared with those of db/m mice (Figure 7G-I), consistent with previous studies 51. Further analysis of the frequency distribution of urine spots revealed that the percentage of small urine spots with an area between 0.1-0.25 cm^2^ (db/m: 15.18 ± 2.35%, db/db: 26.82 ± 4.31%) in the db/db mice was higher than that in the db/m mice (Figure 7J), suggesting that bladder overactivity occurred in the db/db mice. Our results indicated that vesicoureteral reflux and bladder hyperactivity may be involved in the formation and development of hydronephrosis in db/db mice.

## Discussion

In this study, we confirmed that IVCF can well preserve the *in vivo* morphology of the kidneys and that cryo-MOST autofluorescence imaging can be used to obtain 3D images of the whole kidney with its intravital perfusion state and micron resolution. By performing cryo-MOST imaging on UUO, db/db, and control mouse kidneys, we not only observed the distribution of hydronephrosis but also analyzed the changes in multiple renal structures, including renal subregions, arteries, veins, glomeruli, renal tubules, and capillaries. These comprehensive studies of hydronephrosis and its effects on the various structures of the kidneys provide a scientific basis for understanding the pathology and preventing disease-induced injury.

Here, cryo-MOST imaging with IVCF addresses the failure of existing imaging methods to obtain micron-resolution 3D images of whole kidneys under conditions representing *in vivo* perfusion. Traditional whole-kidney imaging methods, such as micro-CT imaging, have a resolution of several to tens of microns, which makes it difficult to correctly distinguish kidney microstructures smaller than 10 μm, such as capillaries 10, 18. In addition, the use of exogenous contrast agents in micro-CT imaging changes the perfusion status of the kidney, and incomplete perfusion of contrast agents may also lead to insufficient detection 10, 52. In contrast, optical imaging has a higher resolution than micro-CT. Previous methods combined with optical clearing 16 or mechanical cutting 53 have achieved submicron-resolution imaging of the whole kidney using dye or transgenic mice. In our work, autofluorescence imaging is achieved by leveraging low temperature and enables the acquisition of a variety of renal anatomical microstructures without any labels. IVCF preserves the *in vivo* perfusion of the kidneys by freezing them in seconds while the mice are alive. Our method can be easily applied to other tissues or organs, including hollow organs (lungs, etc.), liquids (cerebrospinal fluid, hydronephrosis, etc.), soft tissues (kidneys, liver, etc.), hard tissues (teeth, bone, etc.), and even the whole body. Cryo-MOST imaging can also be combined with *in vivo* micro-CT imaging to potentially provide a new dual-mode imaging approach for the study of progressive diseases 29. *In vivo* micro-CT enables the detection of disease progression at multiple time points in the same animal, and cryo-MOST allows the acquisition of high-resolution detailed pathological information at the end time point using the same animal as in *in vivo* micro-CT or at the same time points using different animals. The combination of the two imaging methods allows us to obtain more detailed dynamic and anatomical data to study disease pathogenesis, detect disease progression, and evaluate drug efficacy than can be obtained by a single technique.

Our results demonstrate that volume measurement by 3D whole-organ imaging provides more accurate morphological information for disease-related studies than length measurement by 2D section imaging. For example, compared with the control kidneys, the UUO kidneys showed a decrease in the thickness and increase in the volume of the renal parenchyma and ISOM, suggesting expansion rather than atrophy of the renal parenchyma and ISOM of the UUO kidneys in the early stage. The grading of hydronephrosis, which is of great significance for the judgment of kidney injury and the selection of a treatment plan 9, 40, is generally based on length measurements from 2D sections 54, 55. However, these measurements are greatly affected by the selected position and angle of imaging, so there is no consistent and objective grading method 40. Therefore, we suggest volume measurements as a stable and objective indicator to determine the grade of hydronephrosis and kidney injury. Although 3D volume measurements of the kidney require more work than 2D length measurements, the recent rapid development of deep learning technology has shown great potential for automatic volume identification in various areas of the kidney 56, 57. It can be expected that integrated automated analysis tools can effectively reduce the workload of medical professionals and potentially facilitate the application of 3D measurements in clinical diagnosis 58.

Cryo-MOST autofluorescence images can distinguish the specific morphology of renal tubules, not only enabling us to analyze the diameter and filling status of renal tubules but also providing evidence for the study of related diseases. Renal tubule dilatation is a marker of tubular injury in acute obstructive nephropathy 2, 59 and diabetic nephropathy 60. Our study showed increased PCT and PST diameters in both 3 days post-UUO mice and 15 W db/db mice. However, in cryo-MOST images, abnormal central bulge highlighting was observed in some renal tubules in UUO mouse kidneys but none in db/db mouse kidneys. This difference may be difficult to detect by traditional histology due to the loss of fluid in the renal tubules when sectioning the kidney tissue. Previous studies have shown that the detachment of apoptotic tubule cells in the kidneys of UUO mice leads to occlusion of the tubular lumen, which may be the main cause of abnormal dilatation of some renal tubules, over and above the dilatory effect of hydronephrosis in the renal pelvis 61. This theory may explain the abnormal central highlighting of dilatation in some renal tubule segments found in the UUO mice in our study. In contrast to the UUO model, the dilatation of renal tubules observed in diabetic animals may be due to the increased pressure of tubules caused by polyuria 50 and the dilatation of hydronephrosis, which are less stressful conditions than tubular obstruction. Additionally, the severity of hydronephrosis in the db/db mice was weaker than that in the UUO mice. Perhaps this is the reason why we did not find abnormal urine accumulation in the renal tubules of db/db mice. Therefore, cryo-MOST can not only display changes in renal tubules but also provide clues for exploring the causes of renal tubule lesions.

Measurement of the realistic 3D morphology of renal vessels is essential for the study of oxygen and solute transport 62. However, existing methods cannot accurately display the whole renal vascular network with micron resolution under conditions representing* in vivo* perfusion. This limits the accuracy of computational models of renal oxygenation 37, 62. To our knowledge, this is the first study to demonstrate a micron-scale 3D vascular network under conditions representing *in vivo* perfusion. Our study showed that the renal segmental arteries and veins in the 3 days post-UUO mice were compressed but that the cross-sectional area of the arcuate veins was increased, indicating that normal blood circulation was disrupted by accumulated hydronephrosis. Previous studies have shown that renal ischemia and hypoxia can further lead to renal fibrosis, which is one of the main types of renal damage caused by UUO 63-65. Therefore, our study of blood vessels suggests that more attention should be given to the restoration of renal blood flow and blood pressure when preventing renal fibrosis caused by hydronephrosis in the clinic. In contrast to UUO mouse kidneys, the blood vessels in the db/db mouse kidneys were not different from those in the control kidneys, and only parts of the veins adjacent to areas of hydronephrosis were oblate, indicating that the pressure of hydronephrosis on the renal vessels was mild. However, it should be noted that the severity of hydronephrosis in the db/db mice increased with age (Figure 5, Figure S6). Some studies have shown that hypoxia and renal fibrosis occur in older db/db mice 66, 67. Therefore, the progression of hydronephrosis with age may adversely affect renal blood vessels in the future, which merits attention and further study.

In cryo-MOST images, we can directly measure the functional capillary density due to the autofluorescence absorption of red blood cells 26, 68. The functional capillary density is a useful indicator of the quality of capillary perfusion and tissue oxygen content under both physiological and pathophysiological conditions 69. Therefore, cryo-MOST imaging offers unique advantages for the study of functional capillary injury and hypoxia in the whole kidney. In this study, we found 3 days post-UUO mouse kidneys showed loss of peritubular functional capillaries in the cortex and IM but not in the OSOM. We suspect that there are two reasons for this difference. 1. Compared with the cortex and IM, the OSOM showed a low functional capillary density in normal mice (Figure 4F). Previous studies also showed that OSOM renal tubules were usually in a critical state of hypoxia in normal mouse kidneys 70, and the lower functional capillary density may contribute to the critical state of hypoxia. 2. Kida Y et al. proposed that dilated renal tubules compress the peritubular capillaries, which is related to capillary loss 71. We observed many dilated renal tubules in the cortex of UUO mice, which may be related to the decrease in the functional capillary density. The IM is compressed by the hydronephrosis due to its proximity to the renal pelvis, and we found that the volume of the IM was decreased in UUO mice, which may be related to the decreases capillary density in the IM. In contrast, the volume of the OSOM did not change in UUO mice, and the number of dilated renal tubules was less than that in the cortex and ISOM. Therefore, different strategies should be adopted to treat capillary loss in different subregions. For instance, we may be primarily concerned with the release of hydronephrosis in the IM and to the protection of renal tubules in the cortex. Thus, the comprehensive analysis of various structures of the kidney using cryo-MOST images is conducive to exploring the pathogenesis and developing treatment strategies. In studies on the effect of diabetes on peritubular capillaries, different experimental models are often inconsistent. Maja T Lindenmeyer et al. showed loss of renal interstitial capillaries in human patients with diabetes 72. The results reported by Dominguez, Jesus H. et al. in Zucker and SHHF/GMI-FA hybrid rats showed a 58% reduction in the peritubular capillary density throughout the cortex and medulla in untreated obese rats 73. However, Kosugi, Tomoki, et al. found increased endothelial cells in glomeruli and peritubular capillaries in db/db mice, indicating that diabetes may be related to excessive capillary angiogenesis 74. However, we found that the functional capillary density did not change in 15 W db/db mice. These contradictory findings may be related to the differences in susceptibility and disease development among different animal models 72, 75.

In this study, we did not achieve 3D reconstruction of peritubular capillaries due to the limited Z-axis resolution of 10 or 20 μm. This is because frost caused by low-temperature operation makes it difficult for the cryo-MOST system to achieve long-term continuous imaging at present. In the future, we will further improve the environmental stability and automation of the system to solve this problem. In addition, the clarity of cryo-MOST images is suboptimal due to the use of wide-field imaging. We have previously developed a series of optical sectioning methods based on line scanning to suppress background signals and improve the optical-sectioning ability of high-throughput microscopic imaging 76-79. We will introduce these technologies into cryo-MOST to achieve optical-sectioning imaging. These improvements could potentially facilitate analysis of the 3D spatial relationship between renal capillaries and renal tubules.

The autofluorescence of the renal parenchyma and hydronephrosis observed in this study is mainly derived from FAD 24, 52. We have previously demonstrated that the cryo-MOST system can be used to observe the distribution of autofluorescence of two coenzymes in tissues, FAD and NADH, which are widely used to indicate metabolic status 28, 29. Based on this, we realized the detection of mesoscopic 3D metabolic changes in tumors 29 and whole lungs with acute injury 28 before and after drug treatment and verified the efficacy of drugs against related diseases. Combining these findings with our work here for the 3D reconstruction of blood vessels using FAD autofluorescence imaging, we have the potential to achieve the observation of 3D bivariate maps of blood vessels and metabolism in specific organs or tumors in the future, which could potentially lead to innovative strategies for diagnosis and treatment, such as for the rapid diagnosis of clinical biopsy samples.

## Conclusions

In summary, cryo-MOST imaging with IVCF allows cellular-resolution label-free 3D imaging of hydronephrosis and the renal parenchyma *in vivo*. We evaluated the morphological changes in renal subregions, the 3D vascular network, functional capillaries, glomeruli, and renal tubules in UUO and db/db mouse kidneys and found that the changes were not identical in the two animal models. This method is helpful for understanding the etiology and development of disease and the mechanism of disease-induced injury. Our method is also compatible with fluorescent labels and is potentially applicable to other tissues, organs, and even the whole body.

## Supplementary Material

Supplementary figures.

Supplementary Movie 1. 3D display of C57 mouse kidney.

Supplementary Movie 2. Comparison of serial sections and blood vessels in UUO and control mouse kidneys.

Supplementary Movie 3. Comparison of serial sections and blood vessels in db/db and db/m mouse kidneys.

## Abbreviations

2D: two dimensional
3D: three dimensional
6 W: 6-week-old
8 W: 8-week-old
15 W: 15-week-old
ACR: albumin-to-creatinine ratio
APDRP: anteroposterior diameter of the renal pelvis
Cryo-MOST: cryo-micro-optical sectioning tomography
CT: computed tomography
EVCF: *ex vivo* cryofixation
FAD: flavin adenine dinucleotide
HN: hydronephrosis
IM: inner medulla
ISOM: inner stripe of the outer medulla
IVCF: *in vivo* cryofixation
micro-CT: microcomputed tomography
NADH: nicotinamide-adenine dinucleotide
OSOM: outer stripe of the outer medulla
PAS-stained: periodic acid-Schiff stained
PCT: proximal convoluted tubule
PST: proximal straight tubule
RP: renal parenchyma
UUO: unilateral ureteral obstruction
VSA: voiding spot analysis
