# Supplementary material for: 3D autofluorescence imaging of hydronephrosis and renal anatomical structure using cryo-micro-optical sectioning tomography
Source: Theranostics. 2023 Sep 4;13(14):4885–904. doi: 10.7150/thno.86695 (PMC10526660; doi:10.7150/thno.86695)
Supplement: Supplementary file 1 — Supplementary figures. [file thnov13p4885s1.pdf]

Supplementary Materials

3D autofluorescence imaging of hydronephrosis and renal anatomical structure using cryo-micro-optical sectioning tomography

Guoqing Fan[1], Chenyu Jiang[1], Zhuoyao Huang[1], Mingyu Tian[1], Huijuan Pan[1], Yaru Cao[1], Tian Lei[1], Qingming Luo[1,2,3], and Jing Yuan[1,2,*]

1. Britton Chance Center for Biomedical Photonics, Wuhan National Laboratory for Optoelectronics, MoE Key Laboratory for Biomedical Photonics, School of Engineering Sciences, Innovation Institute, Huazhong University of Science and Technology, Wuhan 430074, China
2. Research Unit of Multimodal Cross Scale Neural Signal Detection and Imaging, Chinese Academy of Medical Sciences, HUST-Suzhou Institute for Brainsmatics, JITRI, Suzhou 215123, China.
3. School of Biomedical Engineering, Hainan University, Haikou, 570228, China.
*Corresponding author: Jing Yuan, E-mail: yuanj@hust.edu.cn.

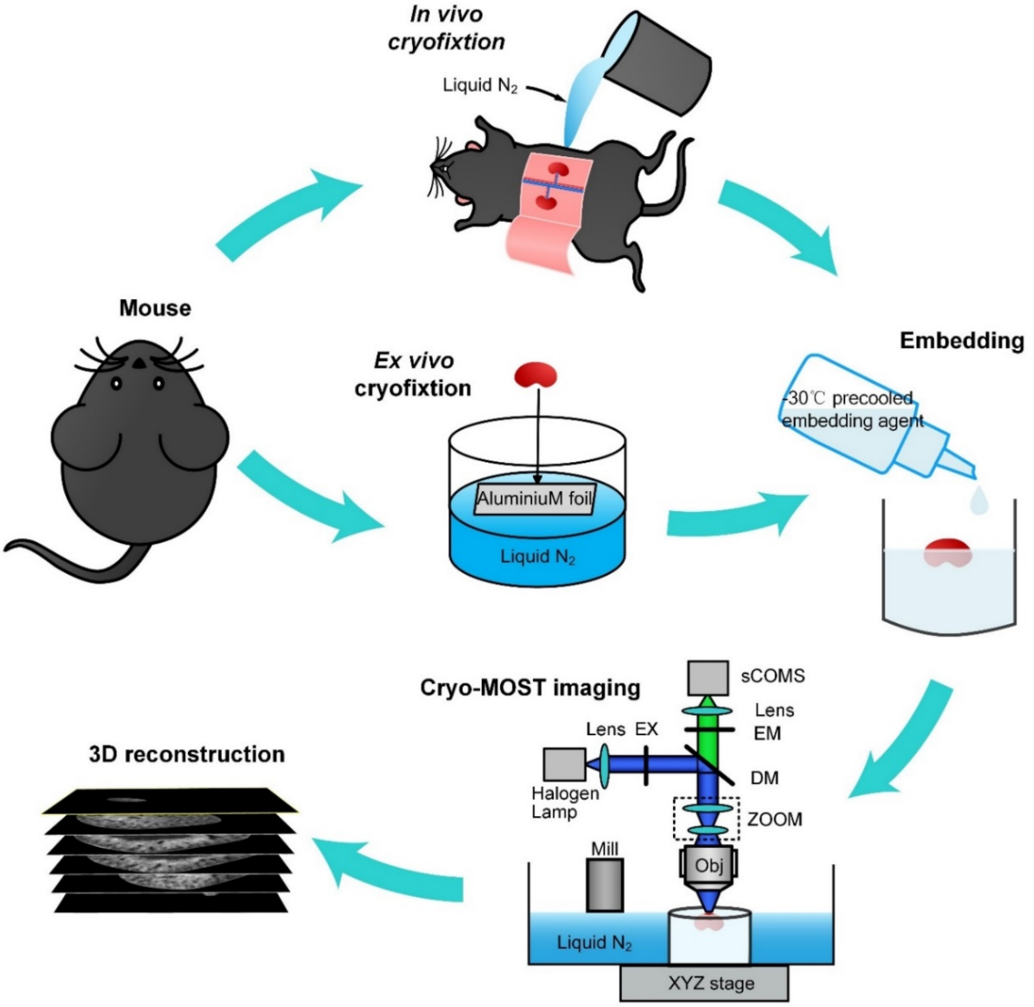

Figure S1. The procedure for *in vivo* cryofixation, *ex vivo* cryofixation, and cryo-micro-optical sectioning tomography (cryo-MOST) imaging. DM, dichroic mirror; Em, emission filter; Ex, excitation filter; Obj, objective lens.

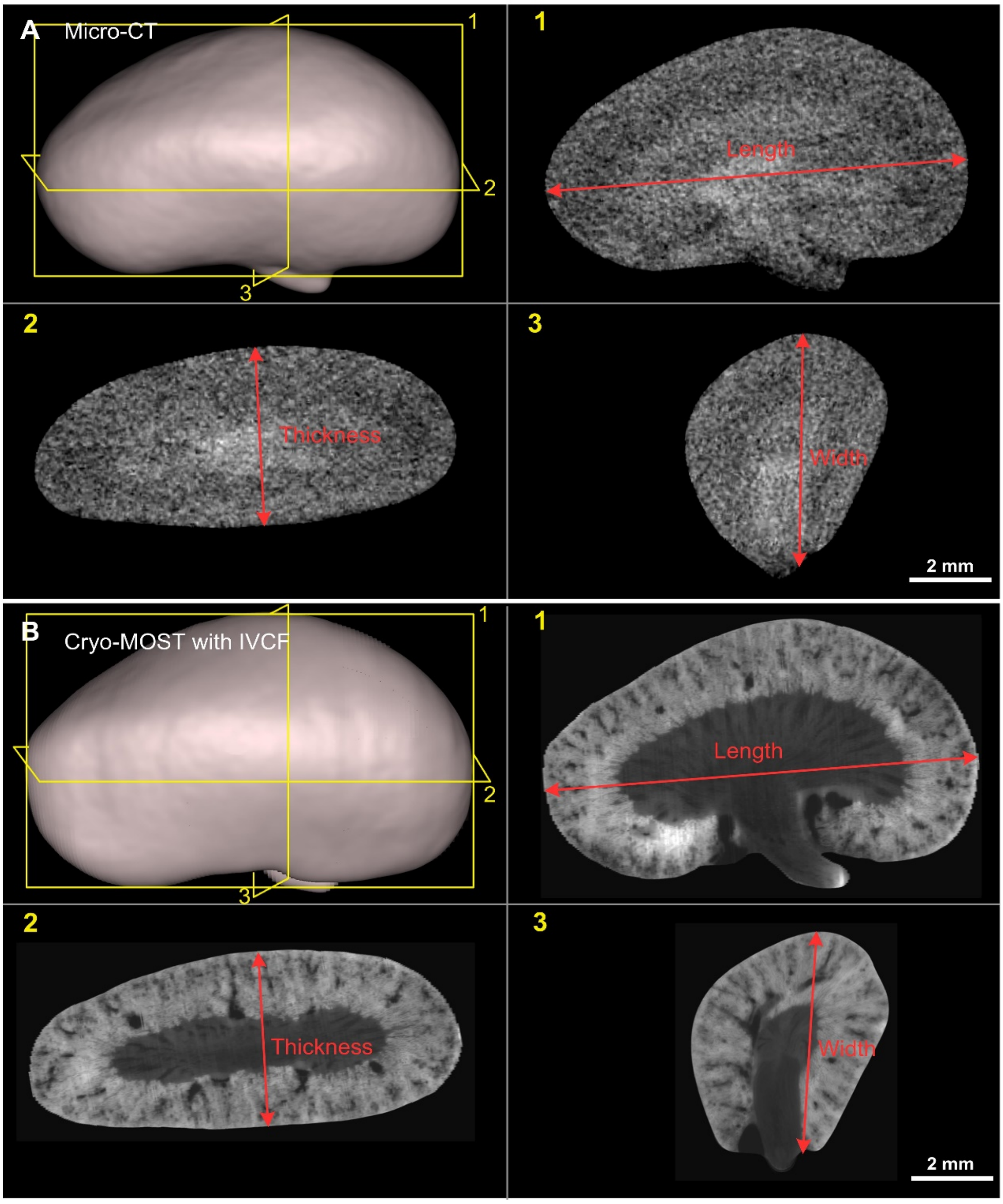

Figure S2. Morphological comparison of the same mouse kidney obtained by micro-CT imaging and cryo-micro-optical sectioning tomography (cryo-MOST) imaging with *in vivo* cryofixation (IVCF). (A) Three-dimensional (3D) reconstruction and three orthogonal sectional images of mouse kidney obtained by micro-CT imaging. 1, 2, and 3 indicate three orthogonal sections. The red bidirectional arrows indicate the length measurement location. (B) 3D reconstruction and three orthogonal sectional images of the same mouse kidney obtained by cryo-MOST with IVCF.

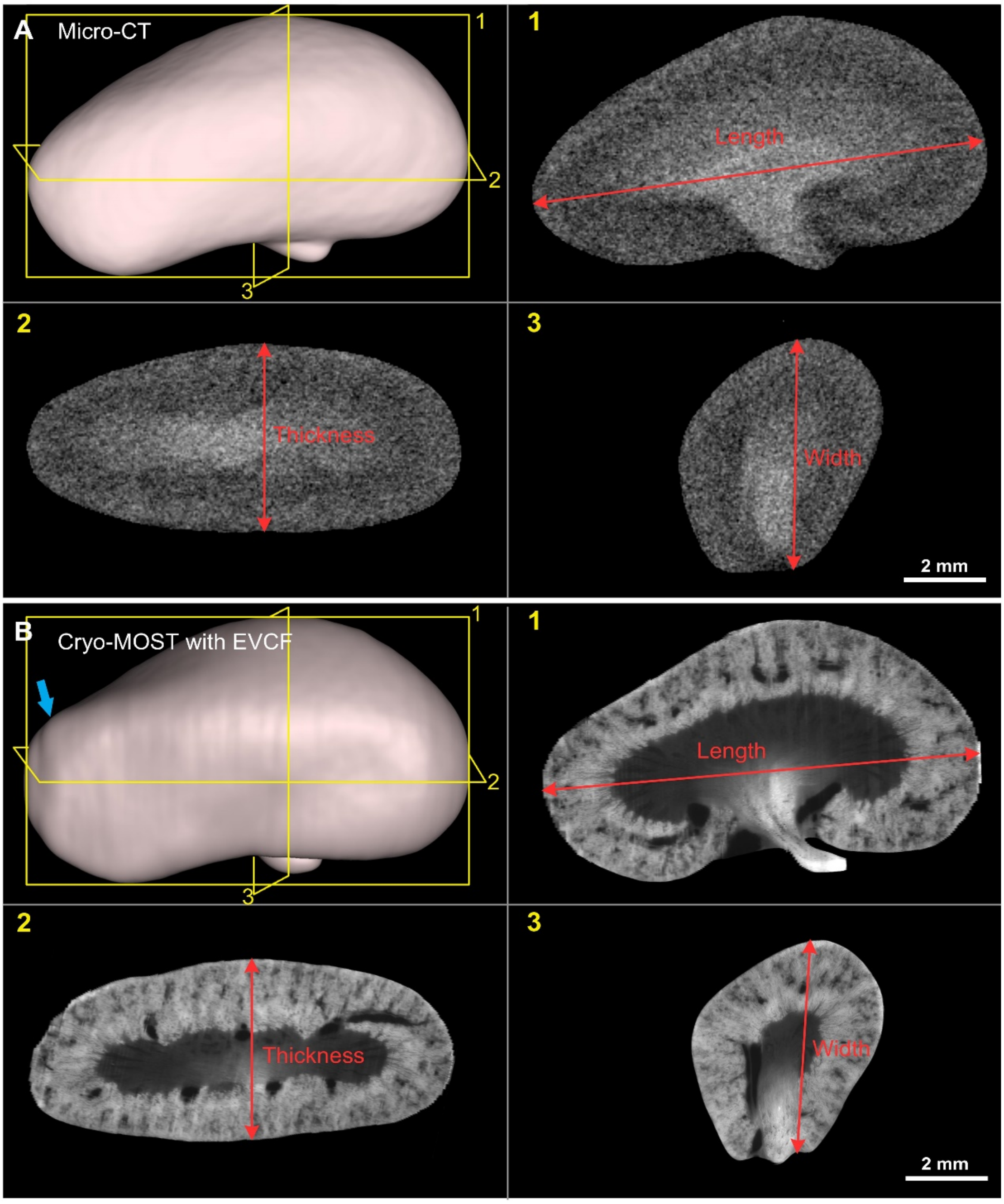

Figure S3. Morphological comparison of the same mouse kidney obtained by micro-CT imaging and cryo-micro-optical sectioning tomography (cryo-MOST) imaging with *ex vivo* cryofixation (EVCF). (A) Three-dimensional (3D) reconstruction and three orthogonal sectional images of mouse kidney obtained by micro-CT imaging. 1, 2, and 3 indicate three orthogonal sections. The red bidirectional arrows indicate the length measurement location. (B) 3D reconstruction and three orthogonal sectional images of the same mouse kidney obtained by cryo-MOST with EVCF.

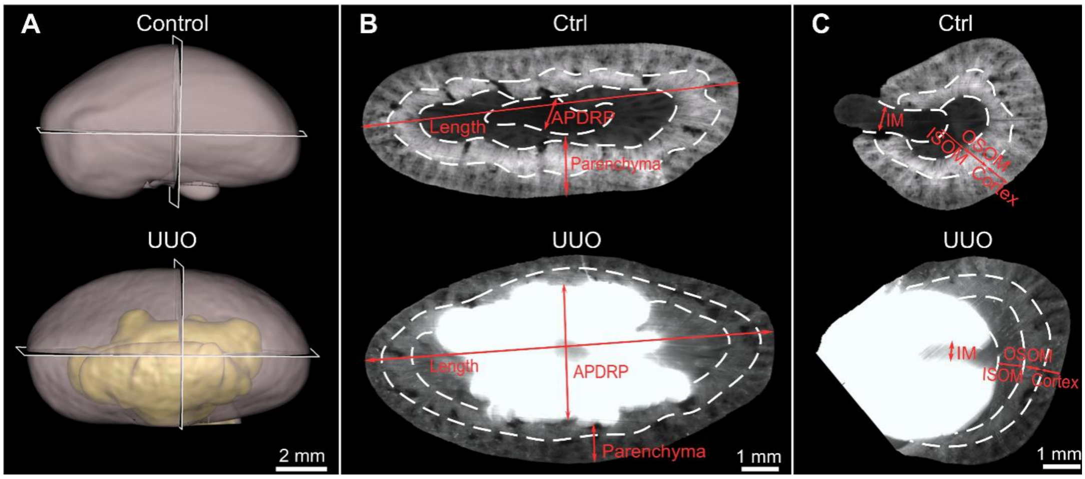

Figure S4. Three-dimensional (3D) reconstruction and section images of control and unilateral ureteral obstruction (UUO) mouse kidneys. (A) 3D reconstruction of control and UUO mouse kidneys. The yellow fill represents hydronephrosis. (B) Sagittal section of control and UUO mouse kidneys. Kidney length, anteroposterior diameter of the renal pelvis (APDRP), and parenchyma thickness were measured as indicated by the red arrow. (C) Transverse section of control and UUO mouse kidneys. The thickness of each renal subregion was measured as indicated by the red arrows.

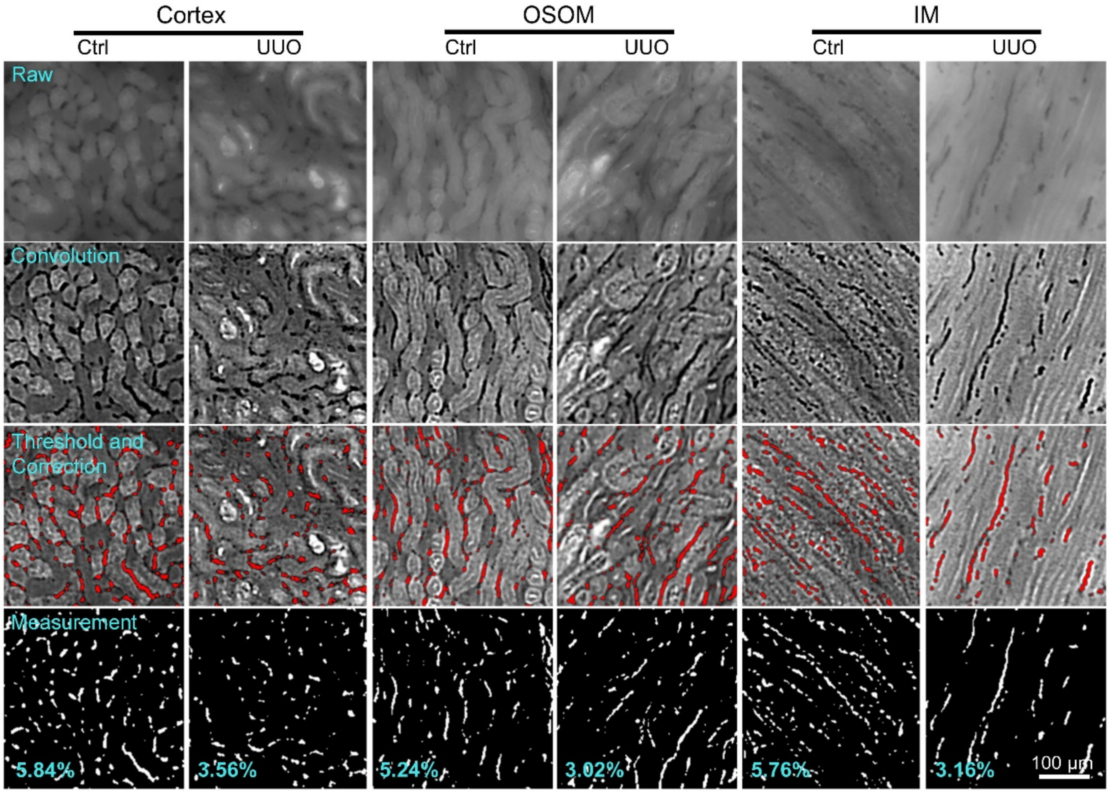

Figure S5. Comparison of functional capillaries in different renal subregions of the control and unilateral ureteral obstruction (UUO) mice. OSOM, outer stripe of the outer medulla; ISOM, inner stripe of the outer medulla; IM, inner medulla.

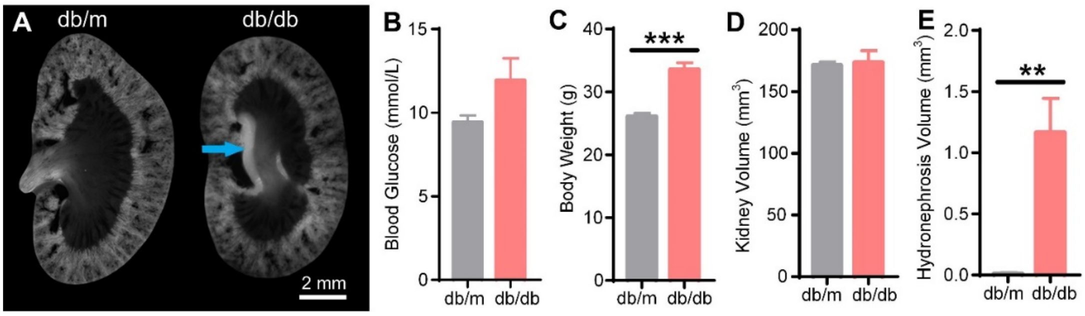

Figure S6. Cryo-micro-optical sectioning tomography imaging and biochemical characterization of 6-week-old db/db and db/m mouse kidneys. (A) Representative coronal section of 6-week-old db/db and db/m mouse kidneys. The blue arrows indicate hydronephrosis. (B) Blood Glucose. (C) Body weight. (C) Kidney volume. (D) Hydronephrosis volume. N = 6 for each group. ** P < 0.01, *** P < 0.001.

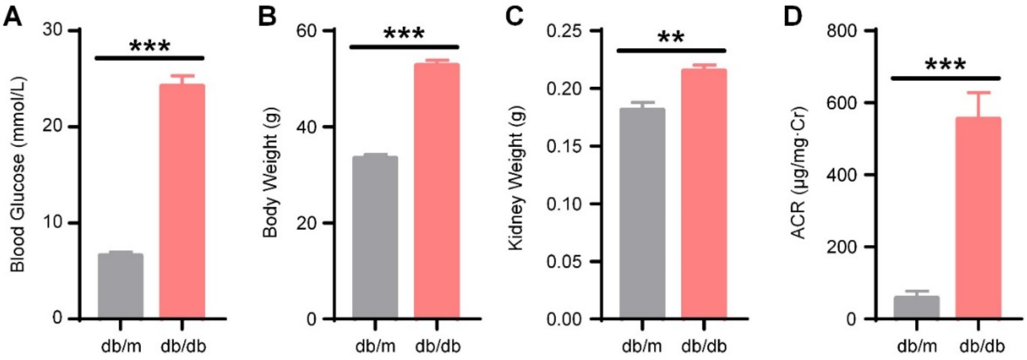

Figure S7. Biochemical characterization of 15-week-old db/db and db/m mice. (A) Blood glucose. (B) Body weight. (C) Kidney weight. (D) Albumin and creatinine ratio (ACR). N = 6 for each group. ** P < 0.01, *** P < 0.001.

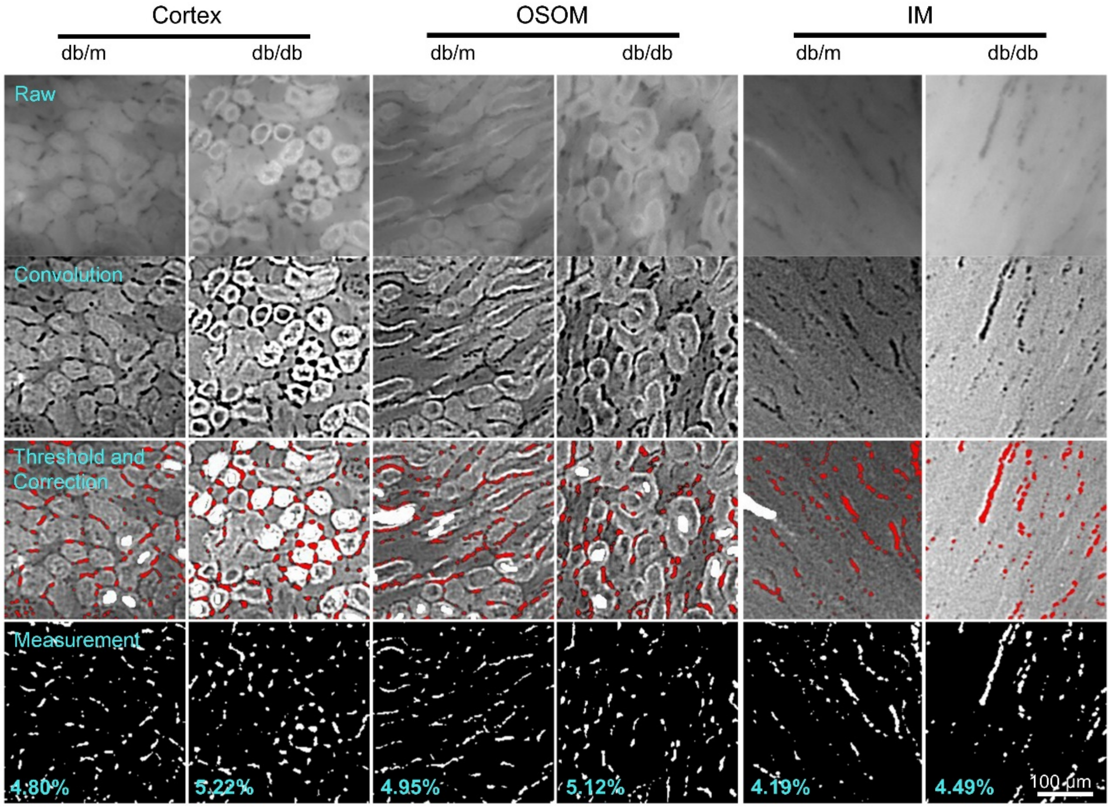

Figure S8. Comparison of functional capillaries in different renal subregions of the 15-week-old db/m and db/db mice. OSOM, outer stripe of the outer medulla; ISOM, inner stripe of the outer medulla; IM, inner medulla.
